# An Apple Susceptibility‐Related LRR Receptor‐Like Kinase MdRLKT21 Activates Plant Immunity by Hijacking a Trans‐Kingdom Fungal microRNA‐Like RNA

**DOI:** 10.1002/advs.202417426

**Published:** 2025-08-04

**Authors:** Mingliang Lei, Jian Zhang, Jie Wang, Chengyu Gao, Yanting He, Runze Tian, Hao Feng, Lili Huang

**Affiliations:** ^1^ State Key Laboratory for Crop Stress Resistance and High‐Efficiency Production College of Plant Protection Northwest A&F University Yangling Shaanxi 712100 China; ^2^ State Key Laboratory of Desert and Oasis Ecology Key Laboratory of Ecological Safety and Sustainable Development in Arid Lands Xinjiang Institute of Ecology and Geography Chinese Academy of Sciences Urumqi 830011 China

**Keywords:** apple, fungal sRNA effector, plant immunity, RLK, Valsa mali

## Abstract

Fungal small RNAs (sRNAs) represent a novel class of effector molecules in plant‐pathogen interactions. However, understanding of how plants counteract fungal sRNAs‐mediated trans‐kingdom RNA interference (RNAi) in plant immunity remains limited. In previous study, a milRNA (*Vm*‐milR1) from *Valsa mali* can be found to transboundary inhibit the expression of disease‐resistance genes *MdRLKT1* and *MdRLKT2* in apple. In this study, a leucine‐rich repeat receptor‐like kinase MdRLKT21 is isolated and characterized, and is confirmed to play a negative regulatory role in the basal immune response. Importantly, MdRLKT21 could hijack *Vm*‐milR1 to rescue the suppression of *MdRLKT1* and *MdRLKT2*, and a similar phenomenon is also observed in the interaction between *V. mali* and pear trees. Meanwhile, it is demonstrated that MdRLKT21 can interact with and phosphorylate a C3HC4‐type RING finger protein MdRFP1, which positively regulates the apple resistance to *V. mali* infection. What's more, it is found that *MdRLKT21* could inhibit the *MdRFP1*‐mediated immune response, probably by promoting the 26S proteasome‐mediated degradation of MdRFP1 in a kinase activity‐dependent manner. Taken together, it is proposed that an apple susceptibility‐related LRR‐RLK competitively binds to fungal sRNA effector and subsequently activates defenses by salvaging the expression of *MdRLKT1* and *MdRLKT2* and thereby releasing the resistance protein MdRFP1.

## Graphical abstract

The apple susceptibility‐related LRR‐RLK MdRLKT21 competitively binds to fungal sRNA effectors and subsequently activates defenses by salvaging the expression of *MdRLKT1* and *MdRLKT2* and thereby releasing the resistance protein MdRFP1. These findings provide new insights into the molecular mechanism by which plants counteract pathogen‐secreted sRNA effectors.

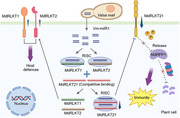

## 1 Introduction

Plants are constantly exposed to diverse microbial organisms, including viruses, bacteria, fungi, and oomycetes.^[^ ^1^ ^]^ To confer full protection against pathogen attack, higher plants possess multiple immune receptors to perceive a variety of immune signals.^[^ ^2^, ^3^ ^]^ Cell‐surface pattern recognition receptors (PRRs) in plants recognize pathogen‐/damage‐associated molecular patterns (PAMPs/DAMPs) and activate pattern‐triggered immunity (PTI).^[^ ^4^ ^]^ This timely recognition triggers a series of key signaling modules, including calcium influx, a burst of reactive oxygen species (ROS), and activation of mitogen‐activated protein kinase (MAPK) cascades.^[^ ^5^, ^6^, ^7^ ^]^ Plant PRR proteins comprise both receptor‐like kinases (RLKs) and receptor‐like proteins (RLPs).^[^ ^6^ ^]^ RLKs contain an intracellular kinase domain, a transmembrane domain, and an extracellular domain.^[^ ^8^ ^]^ Plant RLKs bind to specific ligands through their extracellular domains, including leucine‐rich repeat (LRR), lysin motif (LysM), epidermal growth factor‐like, and lectin‐type domains.^[^ ^8^ ^]^ The largest family of such receptors is known as LRR‐RLKs, which are involved in plant immune responses.^[^ ^9^, ^10^ ^]^ Recent studies have found that most plant LRR‐RLKs could positively regulate immune responses to pathogens. In *Arabidopsis thaliana*, the leucine‐rich repeat receptor‐like kinase FLS2 perceives the bacteria‐derived peptides flg22, triggering the innate immune response.^[^ ^11^ ^]^ Subsequently, the leucine‐rich repeat receptor‐like kinase MIK2 contributes to *Arabidopsis* resistance against *Fusarium oxysporum*.^[^ ^12^ ^]^ In *Nicotiana benthamiana*, a regulator named ELICITIN INSENSITIVE RLK 1 (NbEIR1) enhanced INF1‐triggered immunity and resistance to the model oomycete *Phytophthora capsica*.^[^ ^13^ ^]^ The rice leucine‐rich repeat receptor‐like protein OsRLP1 associates with the receptor‐like kinase OsSOBIR1 to mediate rice defence against a viral pathogen.^[^ ^14^ ^]^ Notably, specific LRR‐RLKs could also act as negative regulators in plant‐pathogen interactions. BIR2, a novel type of LRR‐RLK, has a negative regulatory role on PTI by limiting BAK1‐receptor complex formation in the absence of ligands.^[^ ^15^ ^]^ The maize gene *ChSK1* confers susceptibility to southern leaf blight caused by *Cochliobolus heterostrophus*.^[^ ^16^ ^]^ In rice, RIR1 could represses disease resistance to the Xoo bacterial pathogen.^[^ ^17^ ^]^ Thus, most plant LRR‐RLKs have been shown to play important roles in response to biotic stresses.

To establish a compatible interaction conducive to proliferation, fungi must either avoid triggering PTI or effectively cope with and suppress it. To do so, the fungus secrete an array of virulence proteins, called effectors, which manipulate host cell physiology or suppress plant immune responses to promote colonization.^[^ ^18^ ^]^ These effectors could act either in the extracellular space (apoplastic effectors) to inhibit non‐specific defense mechanisms or within host cells (cytoplasmic effectors) to exert their functions in different organelles.^[^ ^19^, ^20^ ^]^ Consequently, fungal effectors target plant defense responses, cell signaling pathways, and metabolic processes for the benefit of the pathogen.^[^ ^21^ ^]^ A classic example of effectors subverting the host PTI through PAMP manipulation is the LysM domain‐containing effector proteins. The LysM effector *Slp1* (Secreted LysM Protein 1) from *Magnaporthe oryzae* is secreted into the apoplastic spaces, where it binds to chitin released by fungal hyphae, thereby further inhibiting chitin‐induced plant immune responses.^[^ ^22^ ^]^ In monocot rice, different types of viral pathogens directly target OsNPR1 to inhibit OsNPR1‐mediated SA‐JA hormone crosstalk, leading to the cooperatively attenuation of the JA response, thus subverting the rice antiviral immune responses.^[^ ^23^ ^]^ Recent findings have demonstrated that effectors could also target diverse organellar processes of the host. The stripe rust effector *Pst_12806* is translocated to the chloroplast where it interacts with the wheat TaISP protein (a putative component of the cytochrome b6‐f complex). This interaction impairs the photosynthetic process, resulting in less ROS accumulation and ultimately altering host resistance.^[^ ^24^ ^]^ Biotrophic effectors have been known to subvert plant secondary metabolite pathways. In the cytosol of plant cells, the *Ustilago maydis* effector Tin2 carries out the important function by interacting with and stabilizing ZmTTK1 (the maize cytoplasmic protein kinase ZmTTK1), which subsequently enhances anthocyanin biosynthesis.^[^ ^25^ ^]^ Alongside proteinaceous effectors, several nonproteinaceous effectors (NPE) such as small RNA could also hijack the plant immunity system and promote disease progression. Recent studies have shown that plant pathogenic fungi could deliver sRNAs into plant cells, where they act as effectors to silence immune‐related genes by RNA interference (RNAi).^[^ ^26^, ^27^, ^28^ ^]^ During the *Botrytis cinerea*‐*Arabidopsis* interaction, *B. cinerea* sRNAs hijack the plant RNAi machinery by binding to plant Argonaute 1 (AGO1) protein to suppress host immunity.^[^ ^28^, ^29^ ^]^ *Pst*‐milR1, a novel microRNA‐like RNA (milRNA) in *Puccinia striiformis* f. sp. *tritici* (*Pst*), subverts host defense responses by silencing the wheat pathogenesis‐related 2 (*PR2*) gene.^[^ ^30^ ^]^ In addition, *Fol*‐milR1 of *Fusarium oxysporum* f. sp. *lycopersici* (*Fol*) impairs the host immunity machinery by targeting the tomato protein kinase *SlyFRG4*.^[^ ^31^ ^]^

As a consequence of host‐pathogen co‐evolution, plant have developed cytoplasmic nucleotide‐binding leucine‐rich repeat receptors (NLRs) to directly or indirectly detect the more variable effectors, and activate effector‐triggered immunity (ETI), which typically induces a hypersensitive response (HR) and resistance.^[^ ^32^, ^33^, ^34^ ^]^ Intracellular NLRs detect pathogen‐secreted effectors through various mechanisms. In some cases, the modification induced by effectors leads to the destabilization, depletion, or suppression of a host target, and this alteration is subsequently detected. Simultaneously, the host target has evolved to constitutively suppress immune responses, and thus the pathogen‐induced loss of this target could activate the immune response. It now appears that many guarded proteins might actually be decoys, imitating immunity proteins in host defense. These decoys freely evolve to heighten their susceptibility to effector attacks, triggering an immune response. In another variant of ETI, pathogen effector could also induce host cell stress or disrupt homeostasis, which is then detected by the immune system. A simplest version of ETI involves a direct interaction, where the host receptor recognizes the pathogen effector as the specific ligand.^[^ ^35^, ^36^ ^]^ Consequently, some NLRs could perceive a specific effector, whereas others have the capability to detect multiple effectors, and some effectors are recognized by multiple NLRs. Examples include the following: the effector ATR1 from *Hyaloperonospora arabidopsidis* is recognized by the TNL RPP1 in *Arabidopsis* through direct binding.^[^ ^37^, ^38^ ^]^ AvrPphB is recognized by AtRPS5, *Hordeum vulgare* HvPbr1.b and HvPbr1.c.^[^ ^39^, ^40^, ^41^ ^]^ The TNL NbRoq1 recognizes HopQ1‐1, XopQ, and RipB from *Pseudomonas syringae*, *Xanthomonas*, and *Pseudomonas syringae*, respectively.^[^ ^42^, ^43^ ^]^ Despite PTI and ETI are defined as two separate signalling branches, increasing evidence suggests that PRR/co‐receptors are required for full activation of RBOHD during ETI, and ETI triggered by different effectors could potentiate PRR signalling components. Therefore, the two signalling cascades work together in a collaborative manner to ensure effective immunity.^[^ ^44^, ^45^, ^46^, ^47^ ^]^ In addition to the protein effectors, several other non‐proteinaceous pathogen‐derived molecules such as small RNA have also been reported to target multiple host resistance and defense‐related genes. However, the function and molecular mechanism of how plants counteract fungal sRNAs‐mediated trans‐kingdom RNAi in plant immunity remains unclear.

Apple Valsa canker, caused by the ascomycete fungus *V. mali*, poses severe damage and economic losses in apple production, especially in Eastern Asia.^[^ ^48^, ^49^, ^50^ ^]^ Previously, *V. mali* milRNA *Vm*‐milR1 could interfere with the host immunity machinery and promote infection by silencing two host receptor‐like kinase genes *MdRLKT1* and *MdRLKT2*.^[^ ^51^ ^]^ Here, we showed that an apple LRR receptor‐like kinase gene, *MdRLKT21*, competitively attracted fungal sRNA effector *Vm*‐milR1 to rescue the expression of *MdRLKT1* and *MdRLKT2*, and the down‐regulation of *MdRLKT21* could release a disease resistance protein MdRFP1, subsequently activating plant immunity. Collectively, our findings may elucidate the crucial role of the *MdRLKT21* in the response of apples to *V. mali*, and provide new insights into the molecular mechanism by which plant counteract pathogen‐secreted sRNA effectors.

## 2 Results

### 2.1 Overexpression of *MdRLKT21* Suppresses the Plant Immune Responses

Based on the predicted protein structure, MdRLKT21 (GenBank: XP_028946289.1) contained a signal peptide, a LRR domain, a transmembrane domain and a serine/threonine protein kinase domain (Figure S1a, Supporting Information). Multiple sequence alignment revealed that the conserved domains of MdRLKT21 are highly similar among the Rosaceae family (Figure S1b; Table S1, Supporting Information). Phylogenetic analysis demonstrated that *MdRLKT21* has high homology with receptor‐like protein kinase (GenBank: XP_048426566.1) in *Pyrus × bretschneideri* (Figure S1c and Table S1, Supporting Information). To further determine the subcellular localization of MdRLKT21, the fusion protein MdRLKT21‐GFP was transiently expressed in *N. benthamiana*. The green fluorescence of MdRLKT21‐GFP could overlap well with the red fluorescence produced by TaWPI6‐mCherry (a plasma membrane‐located marker protein),^[^ ^52^ ^]^ whereas GFP control was distributed throughout tobacco cells (Figure S2a,b, Supporting Information).The expression of MdRLKT21‐GFP and GFP control were confirmed by Western blotting (Figure S2c, Supporting Information). These results suggest that MdRLKT21 may be a receptor‐like kinase located in the plasma membrane.

**Figure 1:**
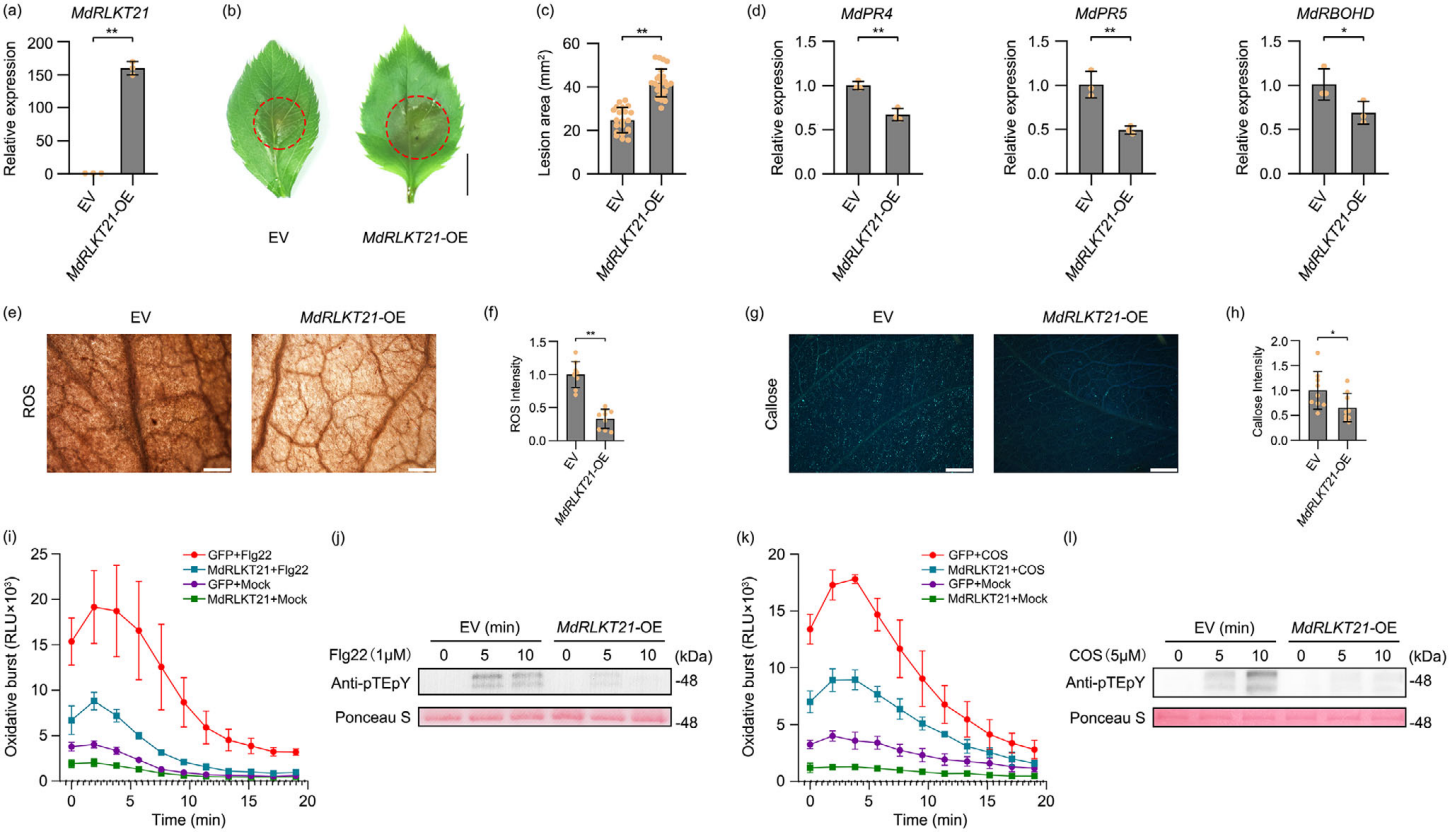
Overexpression of *MdRLKT21* inhibits immune responses in *N. benthamiana* and apple. a) Relative expression levels of *MdRLKT21* in *MdRLKT21*‐overexpressed plants. Mean ± standard deviation (SD) values were calculated from three biological replicates. b,c) The overexpression of *MdRLKT21* significantly reduced resistance to *V. mali* in apple leaves. Photographs were taken at 36 h post *V. mali* inoculation. Lesion areas were measured using ImageJ software. Bar, 5 mm. Mean ± SD values were calculated from at least 21 replicates of three independent biological experiments. d) Overexpression of *MdRLKT21* decreased the expression levels of apple defence‐related genes. *MdEF1α* was used as the inner reference gene. Mean ± SD were calculated from three biological replicates. e,f) Apple leaves overexpressed with *MdRLKT21* showed decreased reactive oxygen species (ROS) accumulation at 36 h post *V. mali* inoculation. Bar, 200 µm. ImageJ software was used to quantify ROS accumulation per microscopic picture. Relative ROS accumulation values were normalized with the mean of empty vector (EV) control. Mean ± SD values were calculated from at least three independent biological replicates. g,h) Apple leaves overexpressed with *MdRLKT21* showed decreased callose deposition at 36 h post *V. mali* inoculation. Bar, 500 µm. ImageJ software was used to quantify callose deposition per microscopic picture. Relative callose deposition values were normalized with the mean of empty vector (EV) control. Mean ± SD values were calculated from at least three independent biological replicates. Statistical analyses were performed using two‐tailed t‐test (^*^ *p* < 0.05 and ^**^ *p* < 0.01). i,k) Influence of flg22‐ and COS‐elicited oxidative burst by *MdRLKT21* in *N. benthamiana*. *MdRLKT21* and GFP control were transiently expressed in *N. benthamiana* leaves. ROS production represented by relative luminescence units (RLU) was measured by treatment with 1µm flg22, 5µm COS, and water. j,l) *MdRLKT21* could suppress the activation of mitogen‐activated protein kinase (MAPK) cascades upon 1 µm flg22 and 5µm COS treatment. Immunoblotting analysis of MAPK with α‐pTEpY antibody. Empty vector (EV)‐treated *N. benthamiana* leaves were used as the control. Proteins were stained with Ponceau S as a loading control.

To investigate whether *MdRLKT21* is involved in host immune responses, *MdRLKT21* was transiently expressed in *N. benthamiana* and apple leaves. The expression level of *MdRLKT21* in the overexpressed apple was significantly upregulated compared with the EV control (**Figure** **1**a). Importantly, the transient expression of *MdRLKT21* enhanced susceptibility to *S. sclerotiorum* and *V. mali* (Figure 1b,c; Figure S3a–c, Supporting Information). Subsequently, the transient expression of *MdRLKT21* significantly decreased the expression of *NbPR4*, *NbPR5* and *NbRBOHB* in *N. benthamiana* leaves, while the overexpression of *MdRLKT21* in apple significantly decreased the expression of the apple defense‐related genes *MdPR4*, *MdPR5* and *MdRBOHD* (Figure 1d; Figure S3d, Supporting Information). Moreover, ROS accumulation and callose deposition in *N. benthamiana* and apple leaves following transient expression of *MdRLKT21* were reduced compared to the control (Figure 1e–h; Figure S3e–h, Supporting Information). The ROS burst assay showed that *MdRLKT21* markedly blocked ROS generation by flg22 and chitosan oligosaccharide (COS), compared to the GFP control (Figure 1i,k; Figure S4, Supporting Information). Immunoblotting analysis using the phospho‐p44/42 MAP kinase antibody showed that *MdRLKT21* could attenuate MAPK activation by flg22 and COS in *N. benthamiana* (Figure 1j,l). These results suggest that *MdRLKT21* suppresses a series of immune responses in *N. benthamiana* and apple.

### 2.2 *MdRLKT21* Negatively Regulates Apple Resistance Against *V. mali*

**Figure 2:**
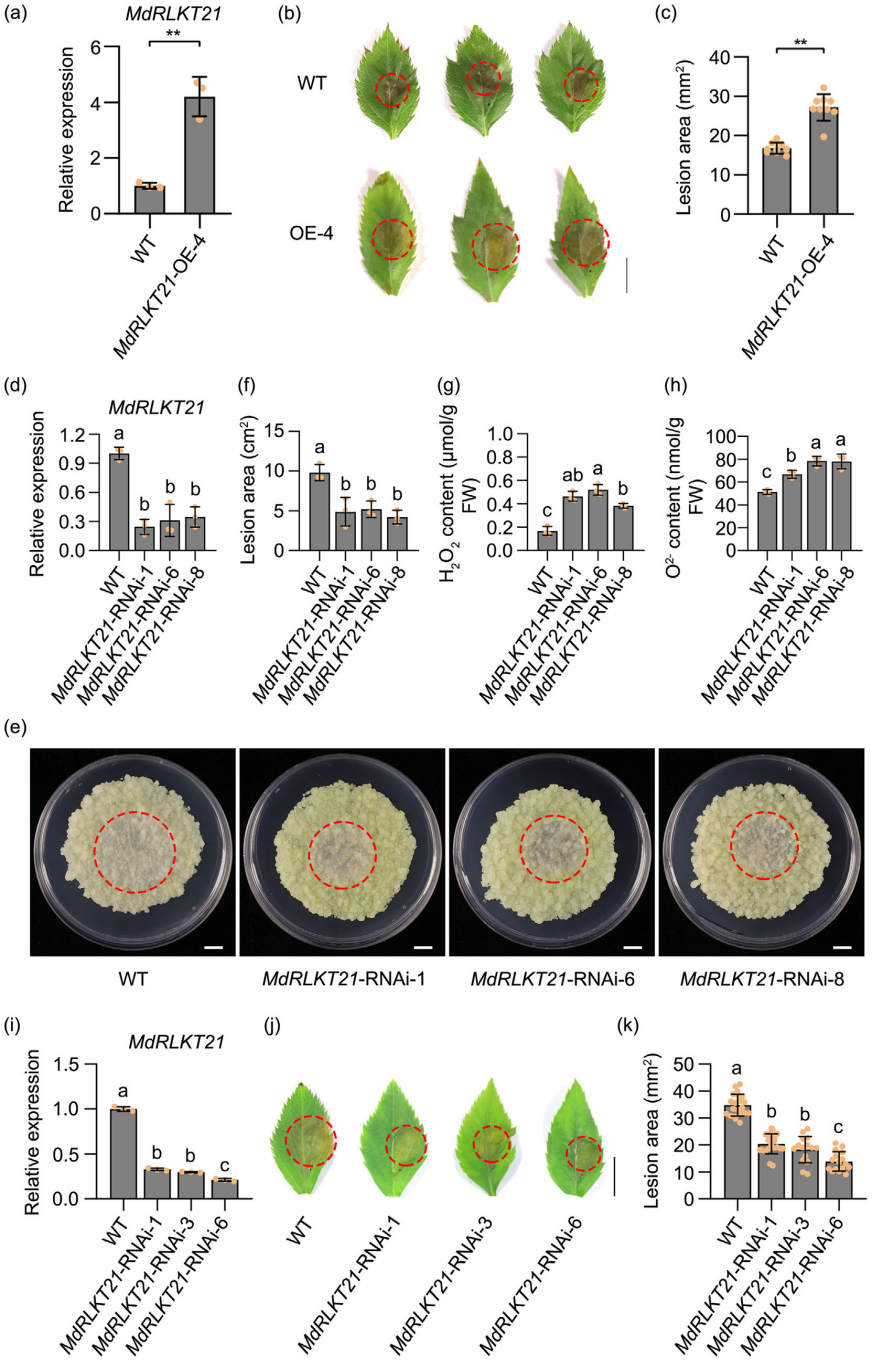
*MdRLKT21* negatively regulates apple resistance to *V. mali* infection in transgenic apples. a) Relative expression levels of *MdRLKT21* in *MdRLKT21*‐overexpressed transgenic apple tissue culture seedlings. *MdEF1α* was used as the inner reference gene. Mean ± standard deviation (SD) values were calculated from three biological replicates. b,c) Stable overexpression of *MdRLKT21* reduced resistance to *V. mali* in apple leaves. Photographs were taken at 36 h post *V. mali* inoculation. Lesion areas were measured using ImageJ software. Bar, 5 mm. Mean ± SD values were calculated from 9 replicates of three independent biological experiments. d) Relative expression of *MdRLKT21* in stable transgenic apple calli. *MdEF1α* was used as the inner reference gene. Mean ± standard deviation (SD) values were calculated from three biological replicates. e,f) Stable silencing of *MdRLKT21* greatly enhanced apple calli resistance against *V. mali*. Photographs were taken at 3 days post *V. mali* inoculation. Lesion areas were measured using ImageJ software. Bar, 10 mm. Mean ± SD values were calculated from three independent biological replicates. g,h). *MdRLKT21*‐silenced apple calli showed increased H_2_O_2_ and O^2−^ contents at 3 days post *V. mali* inoculation. Mean ± SD values were calculated from three independent biological replicates. i) Relative expression of *MdRLKT21* in stable silencing apple seedlings. *MdEF1α* was used as the inner reference gene. Mean ± standard deviation (SD) values were calculated from three biological replicates. j,k) Stable silencing of *MdRLKT21* enhanced resistance to *V. mali* in transgenic apple seedlings. Photographs were taken at 36 h post *V. mali* inoculation. Lesion areas were measured using ImageJ software. Bar, 5 mm. Mean ± SD values were calculated from at least 15 replicates of three independent biological experiments. Two‐tailed t‐test was used for the significant difference analysis (^**^ *p* < 0.01). Different lowercase letters indicate the statistically significant differences (*p* < 0.05, one‐way analysis of variance (ANOVA) and Tukey's multiple comparison test).

To further characterize the molecular mechanism by which *MdRLKT21* compromised disease resistance, we identified three *MdRLKT21*‐OE transgenic apple calli lines, OE‐6, OE‐8, and OE‐11, with *MdRLKT21* expression levels increased by 19.2‐fold, 15.8‐fold, and 22.7‐fold, respectively (Figure S5a,b, Supporting Information). The *MdRLKT21*‐OE‐6/8/11 lines showed larger lesions than the WT upon inoculation (Figure S5c,d, Supporting Information). Furthermore, a stable transgenic seedling overexpressing *MdRLKT21* was generated. Agarose gel electrophoresis experiments demonstrated the presence of specific DNA band in the *MdRLKT21*‐OE line (Figure S6, Supporting Information). RT‐qPCR analysis revealed that the expression levels of *MdRLKT21* was upregulated by 4.2‐fold in the *MdRLKT21*‐OE line compared to the WT (**Figure** **2**a). The inoculation results showed that the lesion areas on the leaves of the *MdRLKT21*‐OE line was significantly larger than the WT (Figure 2b,c). Moreover, the expression levels of the apple defense‐related genes *MdPR4*, *MdPR5*, and *MdRBOHD* were significantly reduced in the *MdRLKT21*‐OE transgenic line (Figure S7, Supporting Information). Light microscopy also revealed extensive colonization of hyphae and disorganized arrangement of plant cells in the *MdRLKT21*‐OE transgenic line (Figure S8, Supporting Information). Furthermore, the leaves of the *MdRLKT21*‐OE line inoculated with the pathogen led to more severe cytoplasmic condensation and chloroplast modification compared to the WT, as observed through transmission electron microscopy (Figure S8, Supporting Information). And the transient silencing of *MdRLKT21* enhances apple resistance against *V. mali* compared to the control (Figure S9a–c, Supporting Information). The expression levels of multiple apple defense‐related genes were increased in apple leaves following transient silencing of *MdRLKT21* (Figure S9d, Supporting Information). Subsequently, we generated three *MdRLKT21*‐RNAi transgenic apple calli lines, RNAi‐1, RNAi‐6, and RNAi‐8, with *MdRLKT21* expression levels reduced by 76%, 72%, and 66%, respectively (Figure 2d; Figure S10a, Supporting Information). Protein abundance detection further confirmed that the RNAi vector was definitely transferred to the apple calli (Figure S10b, Supporting Information). After inoculation, the *MdRLKT21*‐RNAi‐1/6/8 lines exhibited smaller lesions than the WT (Figure 2e,f). Then we examined the accumulation of ROS, the results showed that ROS accumulation was higher in the *MdRLKT21*‐RNAi‐1/6/8 calli (Figure 2g,h). Next, we identified three *MdRLKT21*‐RNAi transgenic apple seedlings, RNAi‐1, RNAi‐3, and RNAi‐6, with *MdRLKT21* expression levels reduced by 67%, 70%, and 79%, respectively, compared to the WT (Figure 2i; Figure S11a–c, Supporting Information). The *MdRLKT21*‐RNAi‐1/3/6 lines exhibited smaller lesions than the WT after inoculation (Figure 2j,k). The expression of *MdRLKT21* was strongly induced (12.2‐fold) in the *V. mali*‐susceptible late‐ripening apple cultivar “Qincui” (Figure S11d, Supporting Information). These results suggest that *MdRLKT21* negatively regulates apple resistance to *V. mali*.

### 2.3 *MdRLKT21* Competitively Attracts *Vm*‐milR1 to Rescue the Expression of *MdRLKT1* and *MdRLKT2*

**Figure 3:**
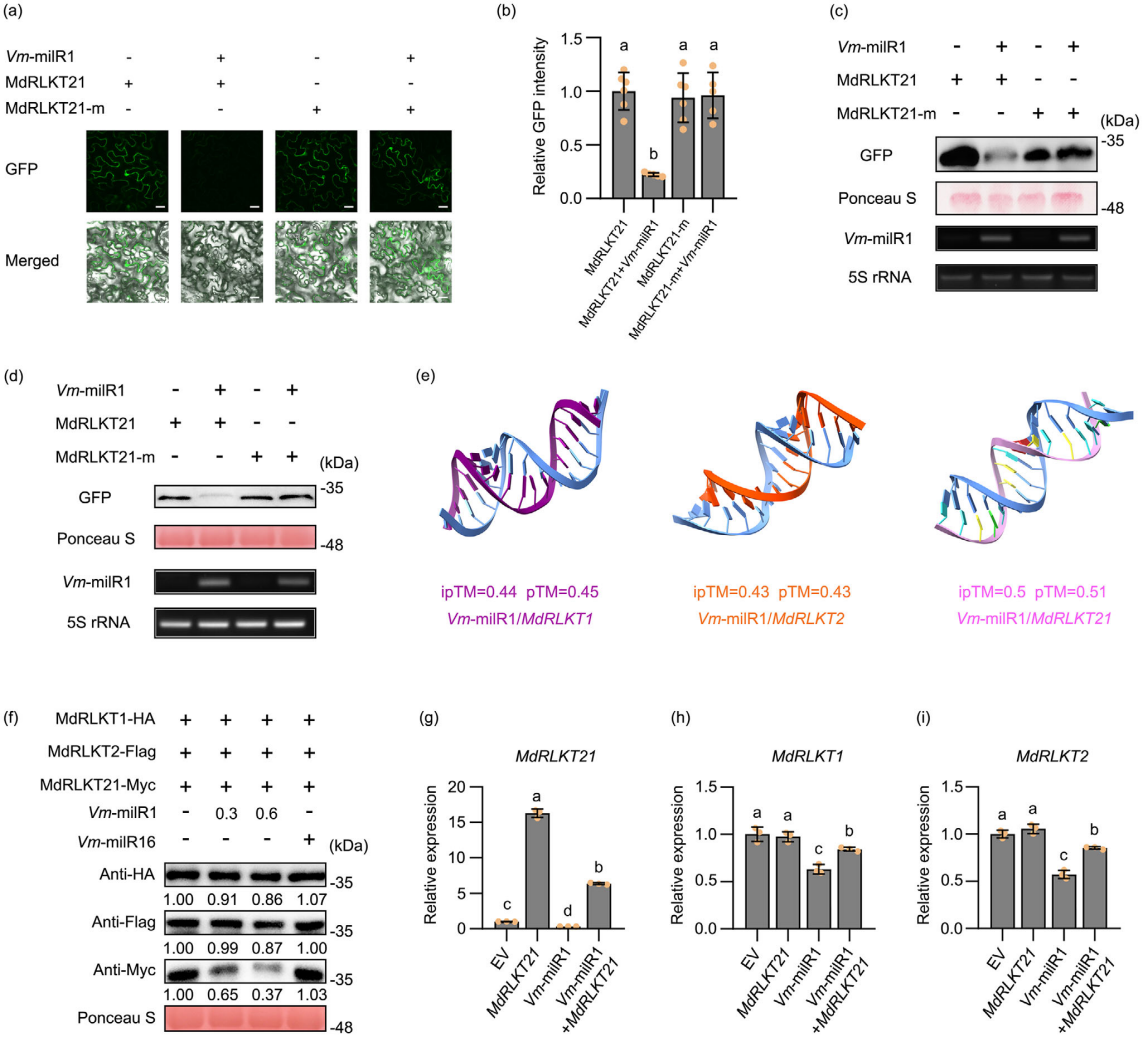
*MdRLKT21* binds more competitively to *Vm*‐milR1 compared to *MdRLKT1* and *MdRLKT2*. a) Co‐expression assays in *Nicotiana benthamiana* revealed that *Vm*‐milR1 silenced *MdRLKT21* in a sequence‐specific manner. *MdRLKT21* target site fused GFP or mutated version (*MdRLKT21*‐m fused GFP) were transiently expressed in *N. benthamiana* leaves with or without *Vm*‐milR1. Green fluorescent protein (GFP) fluorescence was observed at 48 h after agroinfiltration with a laser scanning confocal microscopy using the same parameter settings. Scale bar, 20 µm. b) GFP intensity was determined in at least three independent experiments. Relative GFP intensity was normalized to the mean of GFP intensity of MdRLKT21‐fused GFP. Error bars indicate the SD values. c) Fused GFP was detected by western blotting in *N. benthamiana*. Ponceau S was used as the loading control. Stem‐loop reverse transcription‐polymerase chain reaction (RT‐PCR) was used to detect the expression level of *Vm*‐milR1. 5S rRNA was used as the internal control. Similar results were obtained from three biological replicates. d) Western blotting for fused GFP in apple. Ponceau S was used as control. RT‐PCR was used to detect the expression of *Vm*‐milR1. 5S rRNA as reference. Similar results were obtained from three biological replicates. e) Interaction model prediction between *Vm*‐milR1 and the apple receptor‐like kinase genes *MdRLKT1*, *MdRLKT2*, and *MdRLKT21* based on AlphaFold 3. The accuracy of the prediction could be collectively provided by pTM (Predicted Template Modeling Score) and ipTM (Interface Predicted Template Modeling Score) values. f) Co‐expression assays in *N. benthamiana* showed that *Vm*‐milR1 significantly reduced MdRLKT21 protein levels compared to MdRLKT1 and MdRLKT2. The target sites of MdRLKT1, MdRLKT2, and MdRLKT21 were fused with HA, Flag, and Myc tags, respectively, and co‐expressed in *N. benthamiana* leaves with different concentrations of *Vm*‐milR1. *Vm*‐milR16 was used as negative control. The amounts of proteins were quantified using ImageJ software. Ponceau S was used as the loading control. Similar results were obtained from three biological replicates. g,h,i) Relative expression of *MdRLKT21*, *MdRLKT1*, and *MdRLKT2* in apple leaves were infiltrated the vectors EV, *MdRLKT21*, *Vm*‐milR1, and *Vm*‐milR1/*MdRLKT21*. MdEF1α was used as the inner reference gene. Mean ± standard deviation (SD) values were calculated from three biological replicates. Different lowercase letters indicate the statistically significant differences (*p* < 0.05, one‐way analysis of variance (ANOVA) and Tukey's multiple comparison test).

In the previous study, the *MdRLKT21* expression could be suppressed by *Vm*‐milR1 using both T_ARGET_F_INDER_ and _PS_RNAT_ARGET_.^[^ ^51^ ^]^ To confirm the targeting relationship, the expression levels of *MdRLKT21* was detected during the infection of wild‐type and *Vm*‐milR1 deletion mutant using RT‐qPCR. The expression of *MdRLKT21* was significantly down‐regulated at 6, 12, and 24 hpi in apple leaves inoculated with the wild‐type, but no significant difference during *Vm*‐milR1 deletion mutant infection (Figure S12a,b, Supporting Information). During *Vm*‐milR1 deletion mutant infection, the expression levels of *MdRLKT21* were significantly upregulated compared to infection with the wild‐type (Figure S12c, Supporting Information). Moreover, we conducted co‐transformation assays in *Nicotiana benthamiana* leaves. Compared with the control (*MdRLKT21*‐fused *GFP*), the GFP intensity was greatly suppressed when *MdRLKT21*‐fused *GFP* were co‐expressed with *Vm*‐milR1 (**Figure** **3**a,b; Figure S13a,b, Supporting Information). However, mutated *MdRLKT21* (*MdRLKT21*‐m) could not be silenced by *Vm*‐milR1 (Figure 3a,b; Figure S14a,b, Supporting Information). Western blotting analyses also demonstrated that *Vm*‐milR1 significantly decreased MdRLKT21 protein levels in *N. benthamiana* and apple leaves (Figure 3c,d; Figure S13c, Supporting Information). These results indicate that *MdRLKT21* could be targeted by *Vm*‐milR1 in a sequence‐specific manner.

Since *Vm*‐milR1 could suppress the expression of two disease‐resistant RLKs *MdRLKT1* and *MdRLKT2* according to our previous findings,^[^ ^51^ ^]^ how does *MdRLKT21* participate in the regulation of *MdRLKT1* and *MdRLKT2* by *Vm*‐milR1? First, protein sequence alignment revealed that 29.21%, 26.91%, and 35.18% homology between MdRLKT21‐MdRLKT1, MdRLKT21‐MdRLKT2, and MdRLKT1‐MdRLKT2 pairs, respectively (Table S2, Supporting Information). The biomolecular interaction analysis confirmed that *MdRLKT21* exhibited better binding affinity to *Vm*‐milR1 compared to *MdRLKT1* and *MdRLKT2* using both AlphaFold 3 and _PS_RNAT_ARGET_ (Figure 3e; Figure S15, Supporting Information). To further verify this result, we co‐expressed the vectors MdRLKT1‐HA, MdRLKT2‐Flag, and MdRLKT21‐Myc with *Vm*‐milR1 in *N. benthamiana* and found that *Vm*‐milR1 significantly reduced MdRLKT21 protein levels compared to MdRLKT1 and MdRLKT2, whereas the control *Vm*‐milR16 had no effect (Figure 3f). Furthermore, the apple leaves were infiltrated with various plasmids EV, *MdRLKT21*, *Vm*‐milR1, and *Vm*‐milR1/*MdRLKT21*. *MdRLKT21* and *Vm*‐milR1 were further confirmed to be successfully expressed in various combinations of apple leaves compared to the control (Figure 3g; Figure S16, Supporting Information). The results indicated that transient overexpression of *MdRLKT21* alone did not significantly alter the transcription levels of *MdRLKT1* or *MdRLKT2* compared to the control (Figure 3h,i). However, when *Vm*‐milR1 was overexpressed alone, the transcript levels of *MdRLKT1*, *MdRLKT2*, and *MdRLKT21* were significantly reduced compared to the control (Figure 3g–i). In contrast, co‐expression of *Vm*‐milR1 and *MdRLKT21* led to significant upregulation of *MdRLKT1* and *MdRLKT2* (Figure 3h,i). The LUC assays showed that co‐expression of MdRLKT1/2‐LUC and *Vm*‐milR1 significantly reduced luciferase activity compared to MdRLKT1/2‐LUC alone, whereas the addition of MdRLKT21‐GFP increased the activity (Figure S17, Supporting Information). RT‐qPCR analysis also revealed that the expression levels of *MdRLKT1* and *MdRLKT2* were upregulated in the *MdRLKT21*‐OE transgenic line compared to the WT after inoculation (Figure S18, Supporting Information). These results collectively suggest that *MdRLKT21* could competitively bind to *Vm*‐milR1 compared to *MdRLKT1* and *MdRLKT2*. Actually, *V. mali* was also confirmed as the causal pathogen of pear tree Valsa canker.^[^ ^53^ ^]^ To elucidate whether the mechanism of this competitive combination is conservative, the homologous genes of *MdRLKT2* and *MdRLKT21* in pear (*Pyrus bretschneideri*), which are the probable LRR receptor‐like serine/threonine‐protein kinase (GenBank: XP_048423542.1) and the receptor‐like protein kinase (GenBank: XP_048426566.1), were isolated and used to analyzed the competitive combination between *Vm*‐milR1 and candidate target genes. Based on _PS_RNAT_ARGET_ prediction, *PbRLKT2* and *PbRLKT21* could be targeted by *Vm*‐milR1 at the post‐transcriptional level, and *PbRLKT21* also exhibited a stronger binding affinity to *Vm*‐milR1 compared to *PbRLKT2* (Figure S19, Supporting Information). These results suggest that the phenomenon of small RNA effectors being recognized by host and then throttling competitively may be universal in the *Rosaceae* family.

### 2.4 MdRLKT21 Interacts with and Phosphorylates MdRFP1

**Figure 4:**
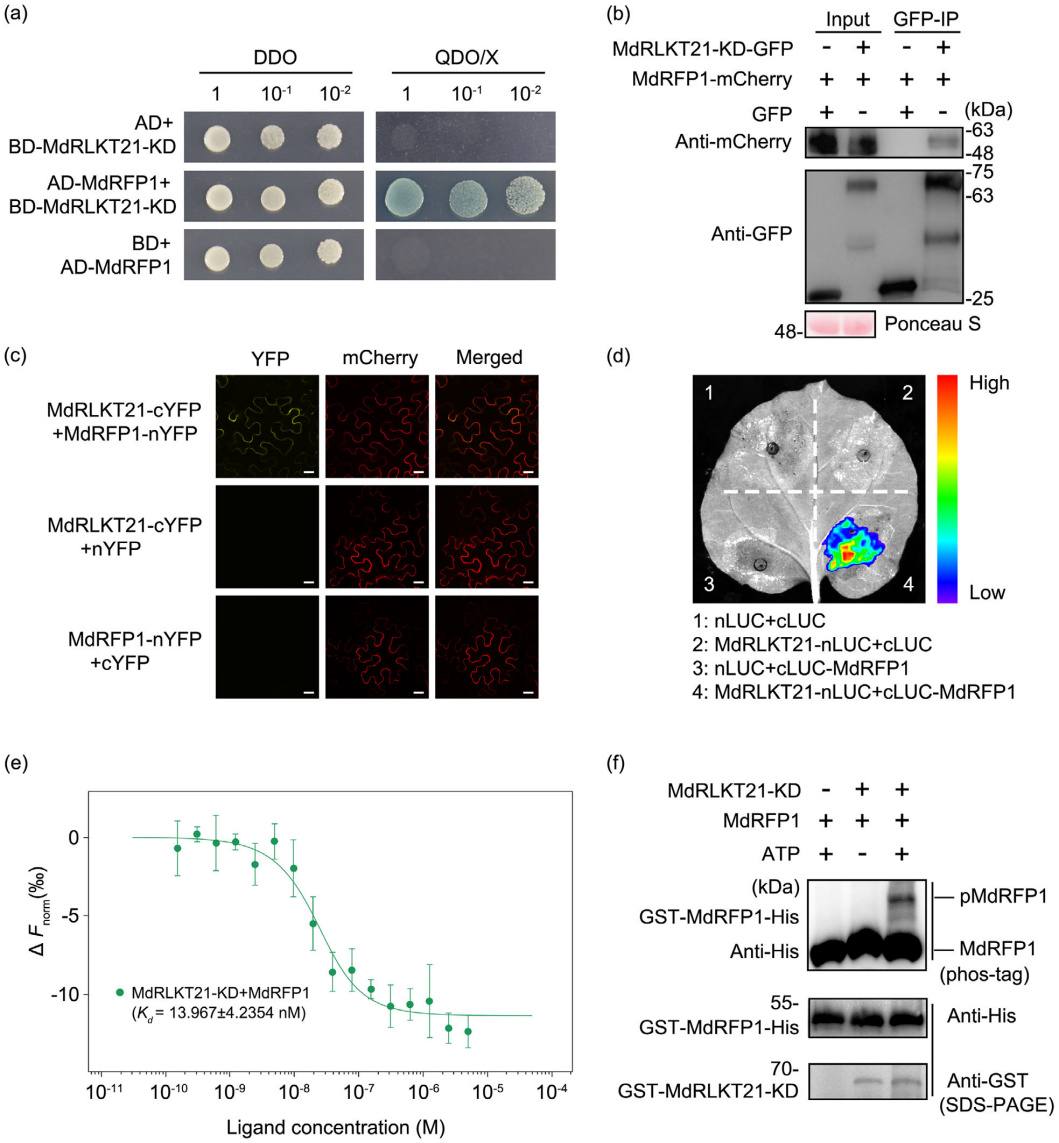
MdRLKT21 physically interacts with and phosphorylates MdRFP1. a) Yeast two‐hybrid (Y2H) analysis indicated that MdRLKT21‐KD interacted with MdRFP1. Yeast strain Y2HGold transformed with the labeled constructs was assayed for growth on SD medium lacking tryptophan and leucine (double dropout [DDO]) and SD medium lacking Adenine, tryptophan, leucine, and histidine with X‐α‐gal (Quadruple dropout [TDO/X]). AD/BD‐MdRLKT21‐KD and BD/AD‐MdRFP1 were used as the negative control. b) Co‐immunoprecipitation (Co‐IP) assay of MdRLKT21‐KD and MdRFP1 interaction. MdRLKT21‐KD and MdRFP1 were co‐overexpressed transiently in *N. benthamiana* leaves. Proteins were immunoprecipitated using GFP‐Trap agarose beads followed by immunoblotting with anti‐GFP and anti‐mCherry antibodies. c) Confirmation of interaction between MdRLKT21 and MdRFP1 using the bimolecular fluorescence complementation assay. cYFP‐MdRLKT21/nYFP and cYFP/nYFP‐MdRFP1 were used as the negative control. Membrane protein TaWPI6 (mCherry‐tagged) was used as the plasma membrane location marker. Scale bar = 20 µm. d) Confirmation of the interaction between MdRLKT21 and MdRFP1 by a luciferase complementation imaging (LCI) assays, the combinations of cLUC with nLUC, MdRLKT21‐nLUC with cLUC, and cLUC‐MdRFP1 with nLUC served as the negative control. e) Binding affinity between MdRLKT21‐KD and MdRFP1 was determined by microscale thermophoresis (MST) analysis. Labeled MdRFP1 was mixed with a gradient dilution of MdRLKT21‐KD recombinant protein. Values represent means±SD from three technical replicates. This experiment was repeated three times with similar results. f) MdRFP1 phosphorylation mediated by MdRLKT21‐KD in vitro. The purified recombinant GST‐MdRLKT21‐KD and GST‐MdRFP1‐His were incubated in protein kinase buffer. Phosphorylation was detected by Phos‐tag SDS‐PAGE.

To elucidate the potential molecular mechanism of *MdRLKT21* in plant immunity, the kinase domain of MdRLKT21 (MdRLKT21‐KD) was used to screen the yeast two‐hybrid (Y2H) library. A C3HC4‐type RING finger protein 1 (MdRFP1) (GenBank: XP_017184215.2) was identified as a potential target of MdRLKT21‐KD. The Y2H assay showed that MdRLKT21‐KD could interact with MdRFP1 in the yeast strain (**Figure** **4**a). The results of the co‐immunoprecipitation (Co‐IP) assays showed that MdRFP1‐mCherry could be detected after MdRLKT21‐KD‐GFP trapping, further confirming the interaction between MdRLKT21‐KD and MdRFP1 in vivo (Figure 4b). Furthermore, the bimolecular fluorescence complementation (BiFC) assay was performed to determine the subcellular localization of interaction between the MdRLKT21 and MdRFP1 in *N. benthamiana*. The YFP fluorescence signals were observed in the cell membrane when MdRLKT21 and MdRFP1 were co‐expressed in *N. benthamiana* cells (Figure 4c). In addition, the luciferase complementation imaging (LCI) assays were performed to verify the interaction. The luciferase activity signals were detected in *N. benthamiana* leaves infiltrated with MdRLKT21‐nLUC and MdRFP1‐cLUC (Figure 4d; Figure S20, Supporting Information). Molecular docking was performed to assess the 3D interaction between MdRLKT21 and MdRFP1. The results indicate that the kinase domain of MdRLKT21 stably binds to MdRFP1 by means of the intermolecular forces formed between multiple key amino acid residues (Figure S21, Supporting Information).

To confirm the MdRLKT21‐MdRFP1 interaction and quantify their binding affinity, we conducted microscale thermophoresis (MST) assays. The GST‐MdRLKT21‐KD and GST‐MdRFP1‐His recombinant proteins were expressed and purified from *Escherichia coli* (Figure S22, Supporting Information). Quantitative MST analysis demonstrated a robust interaction between MdRLKT21‐KD and MdRFP1, with a dissociation constant (*K*_d_) of 13.967±4.2354 nm (Figure 4e). Then, in vitro phosphorylation assay was performed and visualized by Phos‐tag SDS‐PAGE following immunoblot analysis with anti‐His antibody. The result showed that a slower migrating GST‐MdRFP1‐His protein appeared in the presence of adenosine triphosphate (ATP), indicating that MdRLKT21‐KD could phosphorylate MdRFP1 (Figure 4f). These results suggest that MdRLKT21 physically interacts with and phosphorylates MdRFP1 on the cell membrane.

### 2.5 *MdRFP1* Plays a Positive Role in Plant Immunity

**Figure 5:**
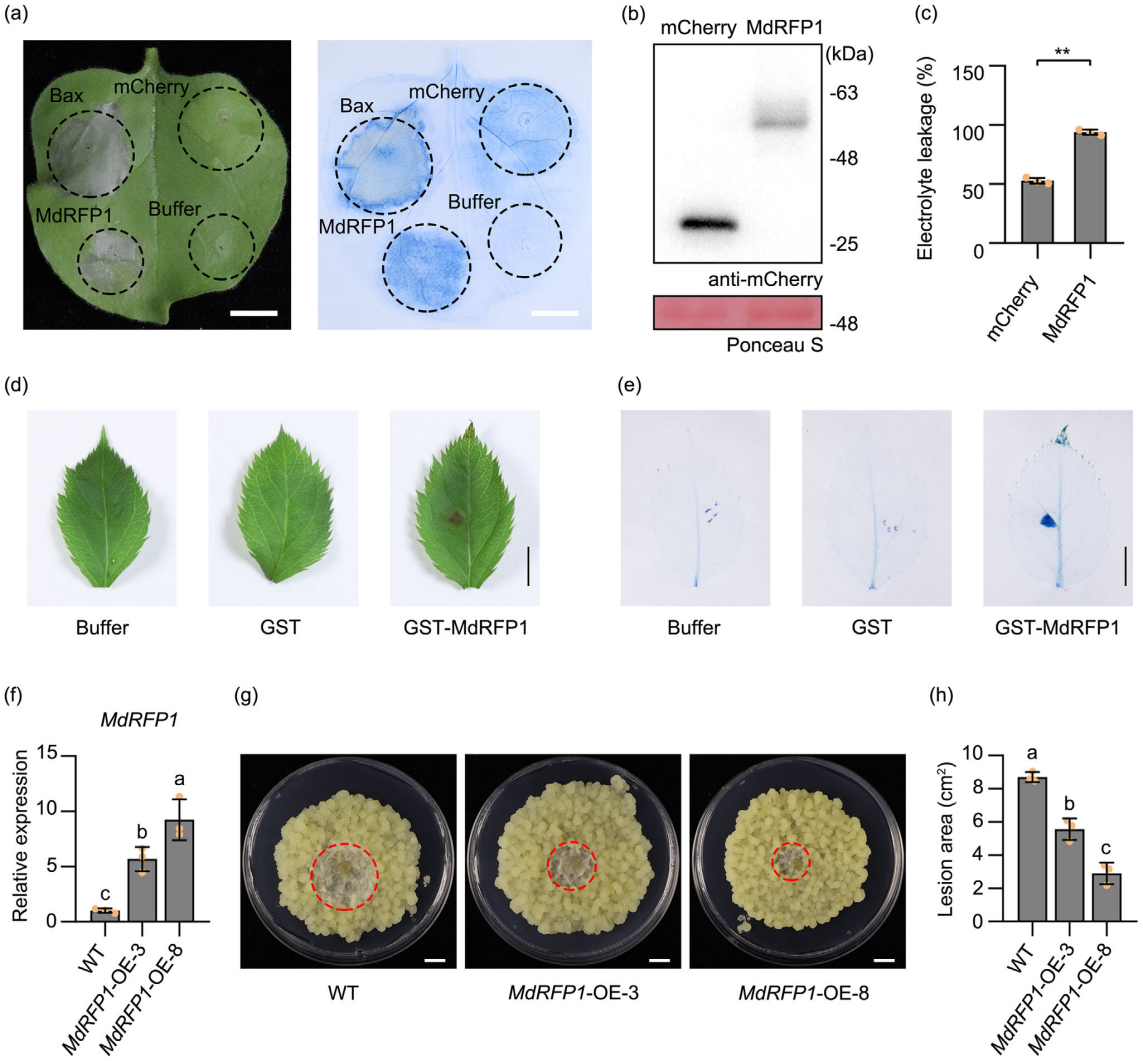
MdRFP1 induces plant cell death and confers apple calli resistance to *V. mali*. a) Representative leaves showing cell death induced by MdRFP1 in *N. benthamiana*. MdRFP1 was transiently expressed in *N. benthamiana* by agroinfiltration. Photographs were taken 5 days post agroinfiltration (dpa). Cell death symptoms were further visualized by trypan blue staining. Bar, 10 mm. b) Immunoblot analysis of proteins in *N. benthamiana* transiently expressing mCherry control and MdRFP1 fused with mCherry tag. Ponceau S was used as the loading control. c) Quantification of cell death by measuring electrolyte leakage. Mean ± SD values were calculated from three independent experiments. d,e) Cell death response in apple triggered by 20 µm purified GST‐MdRFP1 recombinant protein. GST‐MdRFP1 protein was infiltrated in the needle‐pricked area of apple leaves. Photographs were taken 2 days post treatment (dpt) for apple. Cell death symptoms were further visualized by trypan blue staining. Bar, 5 mm. f) Relative expression of *MdRFP1* in stable transgenic apple calli. *MdEF1α* was used as the inner reference gene. Mean ± standard deviation (SD) values were calculated from three biological replicates. g,h) Stable overexpression of *MdRFP1* enhanced apple calli resistance against *V. mali*. Photographs were taken at 3 days post *V. mali* inoculation. Lesion areas were measured using ImageJ software. Bar, 10 mm. Mean ± SD values were calculated from three independent biological replicates. Differences were assessed by two‐tailed t‐test (^**^ *p* < 0.01). Different lowercase letters indicate the statistically significant differences (*p* < 0.05, one‐way analysis of variance (ANOVA) and Tukey's multiple comparison test).

To confirm the function of *MdRFP1* in apple resistance against *V. mali*, we characterized the expression pattern of *MdRFP1* during *V. mali* infection stages. The expression of *MdRFP1* was significantly up‐regulated at 12, 24, 48, and 72 hpi (Figure S23, Supporting Information). Subcellular localization analysis showed that MdRFP1 was mainly localized to the cytoplasmic membrane and partially to the nucleus (Figure S24a,b, Supporting Information). Importantly, it was shown that MdRFP1 induced intense cell death in *N. benthamiana*, whereas mCherry control exhibited no effects (**Figure** **5**a–c). Furthermore, the purified GST‐MdRFP1 recombinant protein could also induce cell death in apple (Figure 5d,e; Figure S25, Supporting Information). These results suggest that MdRFP1 could elicit cell death in multiple plant species.

For further research, we generated two *MdRFP1*‐OE transgenic apple calli lines. Agarose gel electrophoresis experiments demonstrated the presence of DNA band exclusively in *MdRFP1*‐OE lines (Figure S26a, Supporting Information). Western blotting analysis confirmed the detection of MdRFP1‐GFP in *MdRFP1*‐OE lines (Figure S26b, Supporting Information). The transcript levels of *MdRFP1* were upregulated by 5.6‐fold and 9.1‐fold in *MdRFP1*‐OE lines, respectively (Figure 5f). Compared to WT, the lesion areas in *MdRFP1*‐OE lines was significantly decreased at 3 days post inoculation (Figure 5g,h). In addition, ROS contents and O^2−^ production rate in *MdRFP1*‐OE calli were significantly higher than those levels in the WT (Figure S27a,b, Supporting Information). *MdRFP1* was further transiently silenced in apple leaves using agroinfiltration. Silencing of *MdRFP1* significantly increased the lesion areas compared to that of the controls (Figure S28a–c, Supporting Information). Furthermore, ROS accumulation in the *MdRFP1*‐RNAi leaves was lower than the EV control (Figure S28d,e, Supporting Information). These results indicate that *MdRFP1* positively regulates apple resistance to *V. mali*.

### 2.6 MdRLKT21 Could Limit the Immune Response Through Promoting MdRFP1 Degradation

**Figure 6:**
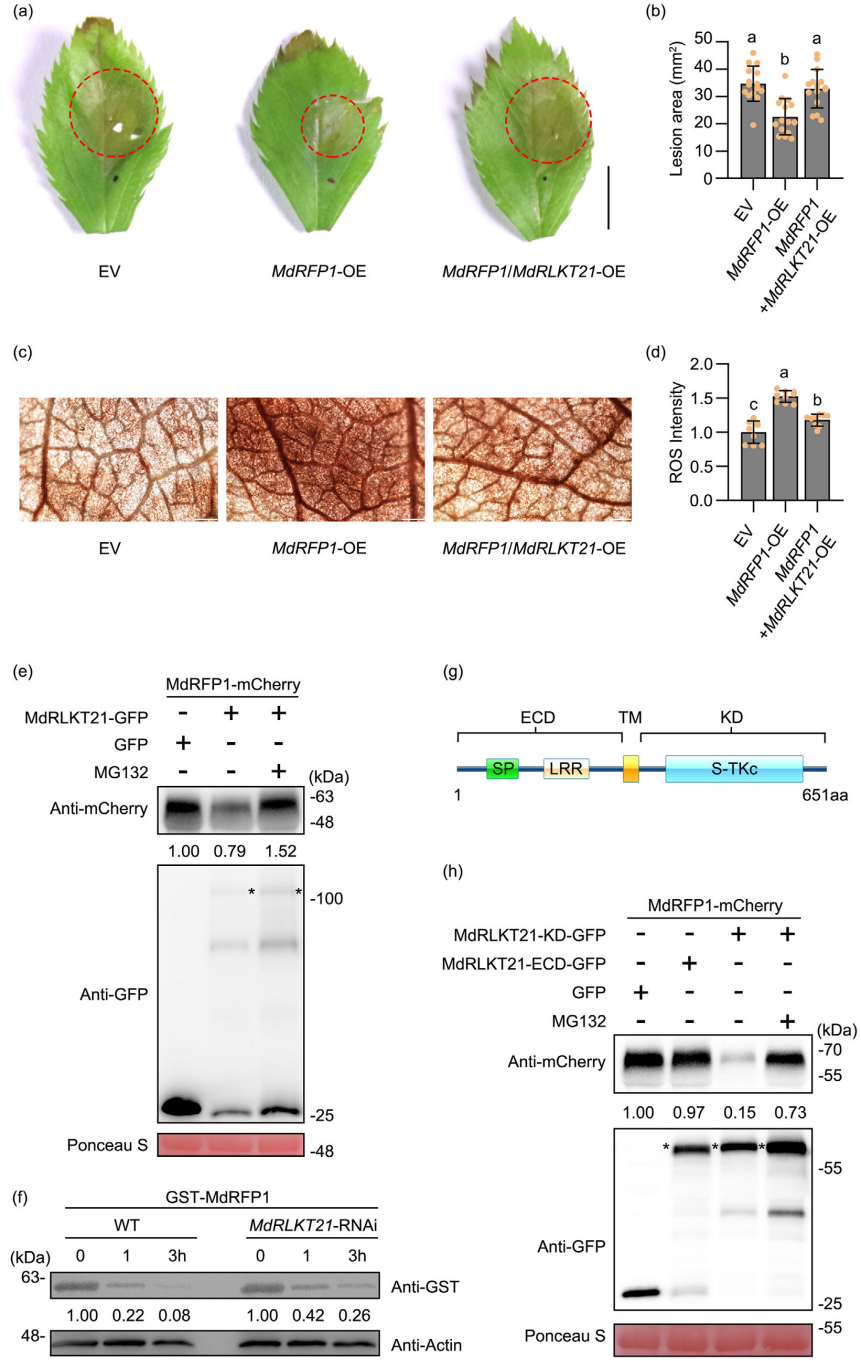
MdRLKT21 inhibits host defence response through promoting MdRFP1 degradation in a kinase activity‐dependent manner. a,b) *MdRLKT21* compromised resistance to *V. mali* in *MdRFP1*‐OE apple leaves. Photographs were taken at 36 h post *V. mali* inoculation. Lesion areas were measured using ImageJ software. Bar, 5 mm. Mean ± SD values were calculated from at least 15 replicates of three independent experiments. c,d) Accumulation of reactive oxygen species (ROS) in apple leaves were infiltrated with the vectors EV, *MdRFP1*‐OE, and *MdRFP1*/*MdRLKT21*‐OE. Bar, 200 µm. Mean ± SD values were calculated from at least three independent biological replicates. Different lowercase letters indicate the statistically significant differences (*p* < 0.05, one‐way analysis of variance (ANOVA) and Tukey's multiple comparison test). e) MdRLKT21 affected the protein stability of MdRFP1 in *N. benthamiana*. Total proteins separately were extracted from *N. benthamiana* leaves expressing the MdRLKT21‐GFP/MdRFP1‐mCherry and GFP/MdRFP1‐mCherry with or without 50 µm MG132. The immunoblotting was performed with anti‐GFP and anti‐mCherry antibodies. The asterisks indicate the position of MdRLKT21 protein. Ponceau S was used as the loading control. f) Protein degradation assays in apple calli. The *E. coli*‐expressed GST‐MdRFP1 protein was incubated with the protein extracts from WT or *MdRLKT21*‐RNAi transgenic apple calli, and then immunoblotting was performed using anti‐GST antibody at the indicated time points. MdActin was used as the loading control. The amounts of proteins were quantified using ImageJ software, and the protein levels at 0 h were set to 1. g) Schematic diagram of the MdRLKT21 protein. The extracellular domain (ECD) encompasses the signal peptide (SP) and leucine‐rich repeat (LRR) domain, while the intracellular kinase domain (KD) comprises the serine/threonine kinase catalytic (S‐TKc) domain. h) MdRLKT21 kinase activity was required for MdRFP1 degradation. Total proteins were extracted from *N. benthamiana* leaves expressing the MdRLKT21‐KD‐GFP/MdRFP1‐mCherry, MdRLKT21‐ECD‐GFP/MdRFP1‐mCherry, and GFP/MdRFP1‐mCherry with or without 50 µm MG132. The immunoblotting was performed with anti‐GFP and anti‐mCherry antibodies. The asterisks indicate the position of MdRLKT21‐KD‐GFP and MdRLKT21‐ECD‐GFP proteins. Ponceau S was used as the loading control.

To investigate the impact of the MdRLKT21‐MdRFP1 interaction on plant immunity, leaves of GL‐3 tissue culture seedlings were transiently infiltrated with the vectors EV, *MdRFP1*‐OE, and *MdRFP1*/*MdRLKT21*‐OE. The expression levels of *MdRFP1* and *MdRLKT21* were significantly increased in apple leaves compared with the EV control (Figure S29a,b, Supporting Information). The inoculation results showed that the lesion areas in *MdRFP1*‐OE apple leaves was significantly reduced compared to the control, while the *MdRFP1*/*MdRLKT21*‐OE leaves displayed more severe symptoms than those of *MdRFP1*‐OE leaves (**Figure** **6**a,b). And ROS accumulation in leaves co‐expressing *MdRFP1*/*MdRLKT21* was lower than that in leaves expressing *MdRFP1* alone (Figure 6c,d). Subsequently, the transient expression of *MdRFP1* significantly increased the expression of *MdPR4*, *MdPR5*, and *MdRBOHD* in apple leaves, while the co‐expression of *MdRFP1*/*MdRLKT21* significantly reduced the expression of these defense‐related genes compared to *MdRFP1* expression alone (Figure S30, Supporting Information). The results indicate that MdRLKT21 may suppress the MdRFP1‐mediated immune response in apple.

We then measured the MdRFP1 protein level after co‐expressing *MdRLKT21* and *MdRFP1* in *N. benthamiana* leaves. The results showed that the protein abundance of MdRFP1‐mCherry was substantially reduced in the presence of MdRLKT21‐GFP compared to the GFP control. However, MG132 could inhibit the degradation of MdRFP1 by MdRLKT21 (Figure 6e). In addition, protein degradation assays in calli showed GST‐MdRFP1 protein levels decreased more slowly in *MdRLKT21*‐RNAi than in WT (Figure 6f; Figure S25, Supporting Information). Then MdRFP1‐mCherry was transiently expressed in WT and *MdRLKT21*‐RNAi‐1/6/8 calli lines, and the amount of MdRFP1‐mCherry was higher in *MdRLKT21*‐RNAi lines compared to the WT (Figure S31, Supporting Information). These results confirm that MdRLKT21 could promote the degradation of MdRFP1 via the 26S proteasome system. Because MdRFP1 encodes a protein containing a C3HC4‐type RING finger domain. The RING finger domain has also been documented with E3 ligase function, which plays a crucial role in ubiquitin‐dependent protein degradation.^[^ ^54^ ^]^ We then measured the MdRLKT21‐KD protein level after co‐expressing MdRLKT21‐KD and MdRFP1 in *N. benthamiana* leaves. The results revealed that MdRFP1‐mCherry, but not the mCherry control, significantly reduced MdRLKT21‐KD‐GFP protein levels, and this degradation was inhibited by MG132 (Figure S32, Supporting Information). The in vivo ubiquitination assays were performed in apple calli. Total proteins extracted from WT and *MdRFP1*‐OE apple calli were incubated with purified MdRLKT21‐KD‐His protein. The ubiquitinated MdRLKT21‐KD‐His protein was analyzed using anti‐His and anti‐Ubi antibodies. A higher quantity of ubiquitinated MdRLKT21‐KD‐His was detected in the MdRLKT21‐KD‐His/MdRFP1‐OE mixture (Figure S33a,b, Supporting Information). The in vitro ubiquitination assays also showed that MdRLKT21‐KD could be directly ubiquitinated by MdRFP1 (Figure S33c, Supporting Information). Therefore, MdRFP1 may exhibit E3 ligase activity and degrade MdRLKT21. Since MdRFP1 is a substrate for phosphorylation by MdRLKT21, we next examined whether the kinase activity of MdRLKT21 affects the stability of MdRFP1. We co‐expressed MdRLKT21‐KD (the kinase domain of MdRLKT21) and MdRLKT21‐ECD (the extracellular domain of MdRLKT21) with MdRFP1 in *N. benthamiana* leaves, respectively. Compared to the GFP control, MdRLKT21‐KD‐GFP significantly reduced the protein abundance of MdRFP1‐mCherry, while MdRLKT21‐ECD‐GFP had little effect. The degradation process was also blocked by MG132 (Figure 6g,h). Collectively, these results illustrated that MdRLKT21 may limit the immune response by promoting MdRFP1 degradation (likely via the 26S proteasome) in a kinase activity‐dependent manner (Figure S34, Supporting Information).

## 3 Discussion

Eukaryotic small RNAs (sRNAs) are noncoding functional regulatory RNA molecules that are 20–30 nucleotides in length.^[^ ^27^ ^]^ sRNAs can be classified as microRNAs (miRNAs) or small‐interfering RNAs (siRNAs).^[^ ^55^ ^]^ Despite their short length, sRNAs exert crucial effects on regulating diverse biological processes in growth, development, metabolism, genome integrity, and immunity.^[^ ^56^ ^]^ Recent studies have provided evidence indicating that sRNAs could travel between hosts and interacting microbes/parasites to silence target genes of diverse kingdoms through a mechanism known as trans‐kingdom RNA interference (RNAi).^[^ ^27^, ^57^ ^]^ Fungal sRNAs represent a novel class of effectors that inhibit host immunity. sRNA effectors were identified from phytopathogenic fungi such as *Botrytis cinerea*, *Verticillium dahlia*, *Fusarium graminearum*, *Hyaloperonospora arabidopsidis*, *Blumeria graminis f. sp. tritici and Fusarium oxysporum f sp. lycopersici*, which were involved in pathogen virulence by silencing the resistance mechanism.^[^ ^28^, ^29^, ^31^, ^58^, ^59^, ^60^, ^61^ ^]^ Trans‐kingdom sRNA transport from microbes to hosts is not restricted to eukaryotic pathogens that encode RNAi machinery, Similar phenomena have been observed in other cases. For example, the parasitic plant *Cuscuta campestris* could send miRNAs into host plants to silence host genes involved in defense responses against *C. campestris*.^[^ ^62^ ^]^ Additionally, the planthopper *Nilaparvata lugens* have evolved insect salivary microRNAs to suppress plant immunity by silencing rice defence‐related gene, *OsbZIP43*.^[^ ^63^ ^]^ Many pathogens could secrete protein effectors into host cells that intercept defence signalling and induce pathogenesis, plants had also evolved intracellular NLRs to detect pathogen‐secreted effectors and trigger immune responses through diverse mechanisms, including the classic guarding decoys model.^[^ ^35^, ^64^ ^]^ However, how plants counteract fungal sRNAs‐mediated trans‐kingdom RNAi in plant immunity remains unclear.

Plant disease susceptibility genes (S‐genes) could be defined as genes that act in a dominant or semi‐dominant manner, and are associated with increased susceptibility or symptoms caused by the pathogen.^[^ ^16^ ^]^ All plant genes that enhance infection and promote compatibility may be regarded as S genes, where mutation or loss of an S gene could limit the capacity of the pathogen to induce disease.^[^ ^65^ ^]^ Previously, the citrus ascorbate peroxidases CsAPX01 and CsAPX02 had been found to confer susceptibility to citrus bacterial canker by regulating hydrogen peroxide levels.^[^ ^66^ ^]^ The photosynthetic gene *OsPsaL* was found to confer host susceptibility to southern rice black‐streaked dwarf virus (SRBSDV) in rice.^[^ ^67^ ^]^ A wheat receptor‐like cytoplasmic kinase gene, *TaPsIPK1*, additionally conferred susceptibility to *Puccinia striiformis* f. sp. *tritici* (Pst).^[^ ^68^ ^]^ The apple FERONIA receptor‐like kinase, *MdMRLK2*, played a negative role in defense against Valsa canker disease.^[^ ^69^ ^]^ Intriguingly, our data demonstrated that *MdRLKT21*‐OE transgenic apple tissue culture significantly increased susceptibility to *V. mali*, while stable silencing of *MdRLKT21* enhanced apple resistance against *V. mali* compared to the control. Clearly, suppression of signaling components in PTI contributes to susceptibility to multiple pathogens, thus genes encoding negative regulators of PTI could also be considered as S genes.^[^ ^65^ ^]^ PTI involves the production of reactive oxygen species (ROS) and the activation of local cell wall fortifications, such as callose deposition.^[^ ^70^ ^]^ ROS could serve as small‐molecule secondary messengers in plant responses to biotic and abiotic stress factors.^[^ ^71^ ^]^ It has long been established that plants respond to pathogen attack with a transient burst of ROS and that ROS play a central role in plant immune responses.^[^ ^72^, ^73^ ^]^ In higher plants, callose is deposited between the plasma membrane and the cell wall at the site of pathogen attack, at the plasmodesmata, and on other plant tissues to slow pathogen invasion and spread.^[^ ^74^ ^]^ In the present study, *MdRLKT21* overexpression lines significantly inhibited *V. mali*‐induced ROS and callose accumulation, and markedly blocked ROS generation by flg22 and COS in *N. benthamiana*. Phosphorylation‐mediated MAP kinase (MAPK) signaling cascades are also well‐known for transducing PTI activation.^[^ ^75^ ^]^ Our data showed that *MdRLKT21* could attenuate MAPK activation in *N. benthamiana*. These results demonstrate that the role of *MdRLKT21* in the susceptibility of apples to *V. mali* may be associated with suppression of the PTI response. In previous study, *Vm*‐milR1 was found to transboundary inhibit the expression of disease‐resistant genes *MdRLKT1* and *MdRLKT2*.^[^ ^51^ ^]^ Further analysis revealed that overexpression of *MdRLKT21* could competitively bind to *Vm*‐milR1 and rescue the expression of *MdRLKT1* and *MdRLKT2*, which appeared contradictory to its potential negative role in plant defense. However, we found that artificial overexpression of *MdRLKT21* could suppress multiple immune responses in both apple and *N. benthamiana*, including ROS production, callose deposition, MAPK signaling pathway, and others. Thus, the slight upregulation of *MdRLKT1* and *MdRLKT2* expression would not completely counteract the suppression of *MdRLKT21* on plant immunity.

Importantly, we also explored the molecular mechanism of *MdRLKT21* in plant immunity, a C3HC4‐type RING finger protein 1 (MdRFP1) was identified as the candidate target proteins of MdRLKT21 using Y2H screening. Zinc finger proteins (ZFPs) are the most abundant class of proteins in eukaryotic genomes, which may contribute to diverse biological functions including DNA recognition, RNA packaging, and lipid binding.^[^ ^76^, ^77^ ^]^ Based on the arrangement of the zinc‐binding amino acids, ZFPs could be classified into the subfamilies of TFIIIA, WRKY, DOF, GATA, RING finger, and PHD.^[^ ^77^ ^]^ The C3HC4‐type RING finger subclass, which is also known as RING‐HC, belongs to the canonical RING finger.^[^ ^78^ ^]^ The C3HC4‐type RING finger protein also seem to be involved in plant defense responses. For instance, overexpressing of *NbZFP1* exhibits significant resistance to tobacco mosaic virus (TMV), cucumber mosaic virus (CMV), and potato virus Y (PVY) in *Nicotiana benthamiana*.^[^ ^79^ ^]^ In particular, OsRHC1 accumulates in the plasma membrane where it enhances the resistance response against *Pseudomonas syringae* pv. *tomato* DC3000.^[^ ^80^ ^]^ The transcripts of *EIRP1* quickly responded to powdery mildew in *Vitis pseudoreticulata* grapevine, and overexpression of *EIRP1* in *Arabidopsis* showed enhanced resistance to the pathogens *Golovinomyces cichoracearum* and *Pseudomonas syringae pv tomato* DC3000.^[^ ^81^ ^]^ In this study, the expression of *MdRFP1* was significantly up‐regulated during *V. mali* infection, suggesting that *MdRFP1* could respond to *V. mali* infection. Furthermore, we found that *MdRFP1* enhanced resistance against *V. mali* in apple calli. Programmed cell death (PCD) is a genetically controlled process that eliminates unnecessary or harmful cells in eukaryotes organisms.^[^ ^82^ ^]^ A widely characterized example of PCD is the hypersensitive response (HR) of plants to invading pathogens, suggesting that PCD could be induced in response to pathogen attack.^[^ ^83^, ^84^, ^85^ ^]^ For example, DAL1 and DAL2, two RING finger proteins homologous to *Drosophila* DIAP1, act as negative regulators of PCD in *Arabidopsis*. In the present study, MdRFP1 exhibited cell death‐inducing activity in *N. benthamiana* and apple leaves, indicating that MdRFP1 might play pivotal roles in the PCD signaling. Ubiquitination, a key post‐translational modification, extensively and precisely modulates plant growth and development, and also plays a central role in plant responses to biotic and abiotic stresses.^[^ ^86^, ^87^ ^]^ And ubiquitination involves tagging target proteins with ubiquitin, leading to their degradation mediated by the 26S proteasome.^[^ ^88^, ^89^ ^]^ For example, a novel MYB TF regulated the cold tolerance and anthocyanin accumulation in association with a bHLH TF and underwent degradation mediated by the 26S proteasome.^[^ ^90^ ^]^ Moreover, an apple BTB domain E3 ligase, MdPOB1, functioned to suppress the apple's defense against the pathogen *Botryosphaeria dothidea* by targeting MdPUB29 for ubiquitin‐mediated proteasomal degradation.^[^ ^91^ ^]^ Phosphorylation can influence the ubiquitination process by regulating the interaction between the substrate and the ligase at the subcellular compartmentalization level.^[^ ^92^ ^]^ OsMPK4 promoted phosphorylation and degradation of IPA1 to confer salt tolerance in rice.^[^ ^93^ ^]^ Mimicking a PBL13 phosphorylated residue at the C‐terminus of RBOHD enhanced PIRE‐mediated ubiquitination during plant immunity.^[^ ^94^ ^]^ A similar molecular mechanism observed in this study, MdRLKT21 suppressed the immune response mediated by MdRFP1, likely through facilitating the degradation of MdRFP1 protein by the 26S proteasome in a kinase activity‐dependent manner. Meanwhile, our results showed that the transcription level of *MdRLKT21* was suppressed by trans‐kingdom *Vm*‐milR1 during *V. mali* infection. Hence, the down‐regulation of *MdRLKT21* could release a disease resistance protein MdRFP1, which subsequently activated plant immunity.

**Figure 7:**
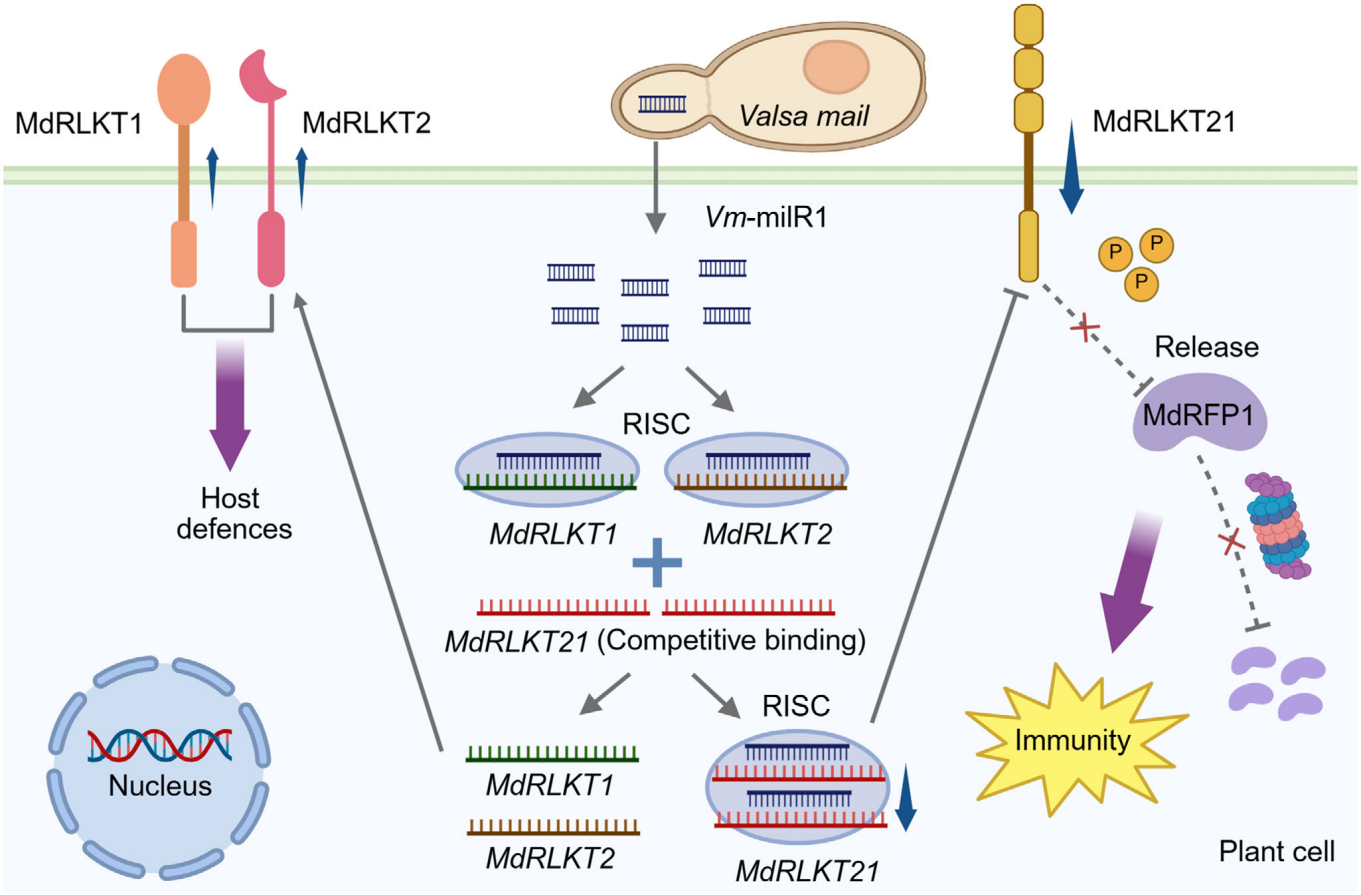
Hypothesis of the action mechanism of *MdRLKT21* during *V. mali*‐apple interaction. In the presence of *V. mali* infection, an apple susceptibility gene *MdRLKT21* competitively binds to trans‐kingdom *Vm*‐milR1 to rescue the expression of disease‐resistance genes *MdRLKT1* and *MdRLKT2*, thereby enhancing apple immunity. Meanwhile, the downregulated *MdRLKT21* could not phosphorylate and degrade MdRFP1, releasing MdRFP1 to further activate the immune response. RISC, RNA‐induced silencing complex.

Susceptibility genes may have been retained through evolution not simply because they are “harmful,” but because they could participate in or influence plant resistance mechanisms under certain circumstances. Therefore, the susceptibility gene *MdRLKT21* might act as a guard decoy, evolving to maximize the susceptibility to sRNA *Vm*‐milR1 attack, which in turn activated immunity through rescuing the expression of *MdRLKT1* and *MdRLKT2* and unleashing the resistance protein MdRFP1 (**Figure** **7**). However, when the susceptible apple cultivar “Qincui” was inoculated, the expression level of *MdRLKT21* was highly induced, suggesting that it might not be completely suppressed by *Vm*‐milR1, leading to further degradation of disease‐resistant proteins. Meanwhile, *MdRLKT1* and *MdRLKT2* were likely targeted by *Vm*‐milR1, ultimately resulting in susceptibility. Nevertheless, our work uncovered a novel regulation mechanism by which a susceptibility gene salvages plant resistance by competitively binding to fungal trans‐kingdom sRNA effector.

## 4 Experimental Section

### Biological Materials and Growth Conditions

The GL‐3 tissue culture seedlings^[^ ^95^ ^]^ were cultivated on Murashige and Skoog (MS) medium containing 0.2 mg L^−1^ 6‐benzyl aminopurine (6‐BA) and 0.2 mg L^−1^ indole‐3‐acetic acid (IAA). *N. benthamiana* seedlings were grown in a phytotron. Plants were grown under a 16‐h light/8‐h dark photoperiod at 25 °C. The apple calli were grown on MS medium containing 0.4 mg L^−1^ 6‐BA and 1.5 mg L^−1^ 2,4‐dichlorophenoxyacetic acid (2,4‐D) at 25 °C in the dark. The *Valsa mali* WT strain 03–8 and *Sclerotinia sclerotiorum* strains were cultured on potato dextrose agar (PDA) medium in a 25 °C incubator.

### RNA Extraction and qRT‐PCR Assays

Total RNA was extracted by the Quick RNA Isolation Kit (Huayueyang) and the miRcute plant miRNA isolation kit (Tiangen) according to the manufacturer's instructions. Stem‐loop reverse transcription‐polymerase chain reaction (RT‐PCR) was performed as described previously.^[^ ^96^ ^]^ First‐strand complementary DNAs (cDNAs) of *Vm*‐milR1 and 5S rRNA were synthesized by miRNA first‐strand cDNA synthesis (Stem‐loop method) (Vazyme) according to the manufacturer's protocol. PCR detection was performed using *Vm*‐milR1‐F and a universal reverse primer. 5S rRNA was used as the inner control.^[^ ^97^ ^]^ To detect the transcriptional level of genes, First‐strand cDNAs were synthesized using the RevertAid First Strand cDNA Synthesis Kit (Thermo Scientific) following the manufacturer's instruction. Quantitative PCR assays were performed using 2 × RealStar Green Power mixture (GenStar) on a LightCycler 96 Real‐Time PCR system (Roche Diagnostics) according to the manufacturer's instructions. *MdEF1α^[^* ^98^ ^]^ and *NbEF1α* ^[^ ^99^ ^]^ were selected as the endogenous control for normalization of gene expression in *M. domestica* and *N. benthamiana*. The results were quantitatively analyzed by the 2^−ΔΔ^ *^C^* ^t^ method.^[^ ^100^ ^]^ The experiments were repeated three times.

### Bioinformatics Analysis

The signal peptide was predicted by SignalP‐5.0 (http://www.cbs.dtu.dk/services/SignalP/). The transmembrane helices in proteins were identified based on TMHMM websites (http://www.cbs.dtu.dk/services/TMHMM/). The protein conserved domains were analysed through the InterPro database (https://www.ebi.ac.uk/interpro/). The protein sequences were obtained from the NCBI database by Blast search programs (https://blast.ncbi.nlm.nih.gov/Blast.cgi). Multiple sequence alignment was performed by ClustalW (https://www.genome.jp/tools‐bin/clustalw), and alignment visualization was performed using ESPript (http://espript.ibcp.fr/ESPript/ESPript/). Phylogenetic trees were constructed using MEGA 11^[^ ^101^ ^]^ and displayed by iTOL (https://itol.embl.de/). The binding efficiency of *Vm*‐milR1 with different *MdRLKs* was predicted using AlphaFold 3^[^ ^102^ ^]^ and _PS_RNAT_ARGET_.^[^ ^103^ ^]^ The 3D structures of MdRLKT21 and MdRFP1 were predicted using SWISS‐MODEL (https://swissmodel.expasy.org/interactive). Molecular docking was then performed using the HDOCK server (http://hdock.phys.hust.edu.cn/), and the resulting complexes were analyzed in PyMOL (version 2.5.5) to evaluate interaction interfaces and binding conformations.

### Co‐Expression of *Vm‐milR1* and Different Target Genes in *Nicotiana benthamiana* Leaves

*Agrobacterium*‐mediated transient co‐expression experiments were performed as described previously.^[^ ^104^ ^]^ Briefly, *MdRLKT21* target region and mutated *MdRLKT21* target region (with a four‐base mutation compared to *MdRLKT21*) were inserted into pCAMBIA1302‐GFP to generate *MdRLKT21‐target region*‐*GFP* and *MdRLKT21‐target region_mut_‐GFP* constructs. These constructs were transformed into *N. benthamiana* leaves with or without Pri‐*Vm*‐milR1 construct^[^ ^51^ ^]^ using *Agrobacterium*‐mediated transformation method. GFP fluorescence was observed with a FV3000 laser scanning confocal microscope (Olympus) at 48 hpi. Proteins were detected with anti‐GFP antibody (Abmart). *MdRLKT1*, *MdRLKT2*, and *MdRLKT21* target regions were fused into pCAMBIA1302‐GFP to generate the *MdRLKT1‐HA‐GFP*, *MdRLKT2‐Flag‐GFP*, and *MdRLKT21‐Myc‐GFP* vectors. These constructs were transformed into *N. benthamiana* leaves with different concentrations of *Vm*‐milR1. *Vm*‐milR1 was set at three different OD_600_ values (0, 0.3, and 0.6), with *Vm*‐milR16 (OD_600_  =  0.6) serving as the control. Proteins were detected with anti‐HA, anti‐Flag, and anti‐Myc antibodies (all from Abmart).

### Genetic Transformation and Pathogen Infection

The overexpression recombinant vectors *MdRLKT21*‐OE and *MdRFP1*‐OE were constructed using the transformation vectors pCAMBIA1302 and pK2GW7. Specific DNA fragments of *MdRLKT21* and *MdRFP1* were cloned into the RNAi vectors pK7GWIWG2D, pART‐CAM, and pFGC5941, respectively. The pK7GWIWG2D and pART‐CAM vectors contained an independent GFP gene, thus allowing the detection of GFP protein in transgenic lines using anti‐GFP antibody. The *Agrobacterium* strains EHA105 and GV3101 (pSoup‐P19) were used for stable and transient expression. These vectors were genetically introduced into GL‐3 tissue culture seedlings, *N. benthamiana* seedlings and apple calli using *Agrobacterium*‐mediated transformation as described previously.^[^ ^105^ ^]^ For pathogen infection assays, the mycelium plugs of *V. mali* were incubated onto leaves of GL‐3 tissue culture seedlings and apple calli. Leaves of *N. benthamiana* seedlings were infected with *S*. *sclerotiorum*. The lesion areas were measured using ImageJ software. Inoculation was performed according to Han's method with the minor modifications.^[^ ^106^ ^]^ The experiments were performed with at least three replications.

### Subcellular Localization of *MdRLKT21* and *MdRFP1*

The coding sequences of *MdRLKT21* and *MdRFP1* were fused to pCAMBIA1302‐GFP vector and transformed into *A. tumefaciens* strain EHA105 by heat shock. *Agrobacterium* with *MdRLKT21‐GFP* and *MdRFP1‐GFP* expression constructs were infiltrated into 4‐week‐old *N. benthamiana* leaves. After 2 days, fluorescence signals were observed using a FV3000 laser scanning confocal microscope (Olympus). The pCAMBIA1302‐GFP empty vector was used as a control. The TaWPI6 protein^[^ ^52^ ^]^ was used as a plasma membrane location marker. Fluorescence intensity was analyzed using ImageJ software. The subcellular localization experiments were repeated three times.

### Light Microscopy and Transmission Electron Microscopy

Samples (1 × 1 × 3 mm) were taken from leaves infection sites at 36 hpi, fixed overnight in 4% glutaraldehyde in PBS at 4 °C, post‐fixed in 1% osmium tetroxide, dehydrated, embedded in LR white resin (London Resin Company Ltd., England), and polymerized at 55 °C. Sections (700 nm semi‐thin, 85 nm ultrathin) were cut using a UC7 ultramicrotome (Leica Microsystems, Germany), stained, and observed under microscopes: semi‐thin with toluidine blue under a BX53/DP70 (Olympus, Japan), and ultrathin with uranyl acetate and lead citrate under a HT‐7700 transmission electron microscope (Hitachi High‐Technologies, Japan) at 80 kV. The methods used were performed as described previously.^[^ ^107^ ^]^

### ROS Detection and Callose Staining

ROS contents and O^2−^ production rate were detected using a hydrogen peroxide assay kit and superoxide anion assay kit (Comin Biotechnology), respectively. ROS staining was performed as previously described.^[^ ^108^ ^]^ The leaves were immersed in 1 mg mL^−1^ DAB solution (pH 3.8) and incubated for 8 h in the light. The leaves were then decolorized with 96% ethanol. Callose staining was performed as previously described.^[^ ^51^ ^]^ The leaves were destained with 96% ethanol and then stained overnight with aniline blue buffer (150 mm K_2_HPO_4_, pH 9.5, 0.01% aniline blue). The accumulation of ROS and callose deposition were counted using the ImageJ software. Photographs were taken using a microscope (Olympus). The experiments were repeated at least three times.

### ROS Burst Measurement and MAPK Assay

Leaf disks were collected from *N. benthamiana* leaves expressing *MdRLKT21* or GFP. The disks were taken in a 96‐well plate and incubated overnight in 100 µL distilled water. Then water was replaced with reaction solution containing 100 µm luminol (Solarbio), 20 µg mL^−1^ peroxidase (Solarbio), and elicitor (1 µm flg22 (Genscript Biotech), 5 µm COS (MedChemExpress), or water as control). Luminescence was quantified utilizing a Varioskan LUX multimode microplate reader (Thermo Scientific). For the MAPK assay, the disks were placed in a 96‐well plate containing 200 µL distilled water overnight, and then treated with 1 µm flg22 or 5 µm COS for 0, 5, and 10 min, respectively. The plant total protein was extracted with native lysis buffer (Solarbio). Proteins were detected using anti‐Phospho‐p44/p42 MAPK antibody (anti‐pTEpY) (Cell Signaling Technology). This assay was performed as described previously.^[^ ^109^ ^]^

### *Y2H*, *Co‐IP*, and *BiFC* Assays

For the Y2H assay, *MdRLKT21‐KD* was cloned into the pGBKT7 vector as a bait, and *MdRFP1* was cloned into the pGADT7 vector as a prey. Various combinations of these vectors were transformed into Y2hGold yeast strain. These yeast cells were grown on SD/–Trp/–Leu (DDO) and SD/–Trp/–Leu/–His/–Ade (QDO) (with X‐α‐gal) plates to verification the interactions. For the Co‐IP assays, *MdRLKT21‐KD* and *MdRFP1* were respectively ligated into pCAMBIA1302‐GFP and PICH86988‐mCherry. The GFP‐Trap beads (Chromotek) were used to separate fusion proteins. This assay was performed as described previously.^[^ ^110^ ^]^ For the BiFC assays, *MdRLKT21* and *MdRFP1* were respectively cloned into pSPYCE and pSPYNE to generate *MdRLKT21*‐cYFP and *MdRFP1*‐nYFP constructs. The constructs were transformed into *Agrobacterium* strain EHA105 and coexpressed in *N. benthamiana* leaves. The signals were observed under a FV3000 laser scanning confocal microscope (Olympus) at 48 h after agroinfiltration. Assays were repeated at least three times.

### LUC Assays and Protein Purification

The ORF sequences of *MdRLKT21* and *GFP* were cloned into pCAMBIA1300‐nLUC vector, and the ORF sequences of *MdRFP1* and *GFP* were inserted into pCAMBIA1300‐cLUC vector. *MdRLKT1* and *MdRLKT2* target regions were cloned into modified pGreenII‐0800‐LUC to generate *MdRLKT1‐LUC* and *MdRLKT2‐LUC* constructs. The constructs were transformed into *Agrobacterium* strain EHA105 and co‐infiltrated into *N. benthamiana* leaves. D‐Luciferin (Solarbio) was used for the LUC imaging. The luciferase expression signals were detected using the PlantView100 multispectral dynamic fluorescence microscopic imaging system (Biolight Biotechnology). Dual Luciferase Reporter Gene Assay Kits (Yeasen Biotechnology) and Varioskan LUX multimode microplate reader (Thermo Scientific) were used for Luciferase activity analysis. The activity was determined as the ratio of firefly luciferase activity to renilla luciferase activity. The coding sequences of full‐length *MdRFP1*, *MdRFP1‐His*, and *MdRLKT21‐KD* were cloned into pGEX‐6P‐1 (with a GST tag), while *MdRLKT21‐KD* was additionally cloned into pCold‐TF (with a His tag). The constructs were transformed into the BL21 (DE3) *E. coli*. The recombinant proteins GST‐MdRFP1, GST‐MdRFP1‐His, GST‐MdRLKT21‐KD and MdRLKT21‐KD‐His were purified using glutathione agarose and HisPur Ni‐NTA resin (Thermo Scientific) following the manufacturer's instructions.

### Microscale Thermophoresis Assay

MdRFP1 was purified and labeled with the Monolith NT Protein Labeling Kit RED (Nanotemper Technologies, Germany; https://nanotempertech.com/) according to the instructions provided by the manufacturer. Labeled proteins was incubated with a gradient dilution of MdRLKT21‐KD recombinant protein for 30 min at RT. Standard treated capillaries (Nanotemper Technologies) were loaded and the measurements were performed via Monolith NT.115 (Nanotemper Technologies). Similar results were obtained from three biological replicates, and data were analyzed by MO. Affinity A_NALYSIS_ v2.3.0 software.

### In Vitro Phosphorylation Assay

Recombinant GST‐MdRLKT21‐KD and GST‐MdRFP1‐His protein were expressed in *E. Coli* BL21 (DE3). GST‐MdRLKT21‐KD protein was incubated with GST‐MdRFP1‐His protein in kinase buffer (50 mm HEPES pH 7.5, 5 mm MgCl_2_, 1 mm DTT, 0.5 mm ATP) at 30 °C for 1 h, and the reaction was stopped by heating at 95 °C for 5 min with SDS loading buffer. The phosphorylated proteins were separated by Phos‐tag SDS‐PAGE and analyzed by western blotting with the corresponding antibodies.^[^ ^68^ ^]^

### Detection of Cell Death in *N. benthamiana* Leaves

Cell death symptoms in *N. benthamiana* leaves were detected by trypan blue staining and ion leakage assays. For apple leaves, cell death induced by the GST‐MdRFP1 recombinant protein was monitored at 2 days post infiltration (dpi) using trypan blue staining. Trypan blue staining was performed based on the method.^[^ ^111^ ^]^ The whole leaves were boiled in the trypan blue solution for 3 min and further incubated for at least 12 h. Then the leaves were destained in a chloral hydrate solution. Measurement of electrolyte leakage was conducted following the description.^[^ ^112^ ^]^ Six disks (1 cm diameter) from agroinfiltrated *N. benthamiana* leaves were soaked in 5 mL distilled water for 5 h at room temperature, and the conductivity (E1) was measured with a conductivity meter (Mettler‐Toledo). Then the leaf disks were boiled for 20 min and the conductivity (E2) was measured again after cooling. The conductivity was determined by the formula (E1/E2) × 100. The experiments were repeated at least three times.

### Protein Degradation Assays

Protein degradation assays were performed according to the method previously reported.^[^ ^113^ ^]^ The total proteins of WT and *MdRLKT21*‐RNAi transgenic apple calli were extracted with degradation buffer containing 25 mm Tris–HCl, pH 7.5, 10 mm NaCl, 10 mm MgCl_2_, 4 mm PMSF, 5 mm DTT and 10 mm ATP. The purified GST‐MdRFP1 protein was added to the equal total protein solution at 23 °C. The samples were harvested at the indicated time. The protein samples were immunoblotted using anti‐GST or anti‐Actin antibodies (Abmart).

### Ubiquitination Assay In Vivo and In Vitro

In vivo ubiquitination assays were performed as previously described.^[^ ^114^ ^]^ Briefly, The WT and MdRFP1‐OE apple calli were collected for protein extraction after treatment with 50 µm MG132 for 8 h. The extracts were incubated with MdRLKT21‐KD‐His protein at 30 °C for 12 h. The total proteins were immunoprecipitated with the anti‐His antibody (Beyotime). The eluted proteins were analyzed by immunoblot with anti‐His and anti‐Ubi antibodies (Abmart). In vitro ubiquitination assays were performed by following the method described previously.^[^ ^91^ ^]^ In brief, UBE1 (E1; Boston Biochem), UbcH5b (E2; Boston Biochem), ubiquitin (Ubi; Boston Biochem), MdRLKT21‐KD‐His and GST‐MdRFP1‐His were mixed in reaction buffer [50 mm Tris‐HCl (pH 7.4), 5 mm MgCl_2_, 100 mm NaCl and 5 mm ATP]. The reactions were incubated at 30 °C for 8 h, and the ubiquitinated MdRLKT21‐KD‐His was detected with anti‐His antibody (Abmart).

### Statistical Analysis

Each experiment was replicated at least three times. Experimental results were analyzed using GraphPad Prism 8 or SPSS software. Significant differences were evaluated through one‐way ANOVA or Student's *t*‐test (*p* < 0.05 and *p* < 0.01). The data were shown as mean ± standard deviation (SD).

## Conflict of Interest

The authors declare no conflict of interest.

## Author Contributions

L.H., H.F., and M.L. designed the study. M.L., J.Z., J.W., and R.T. performed the experiments. M.L., C.G., and Y.H. analyzed the data. M.L., H.F., and L.H. wrote the manuscript.

## Supporting information



Supporting Information

Supporting Information

## Data Availability

The data that support the findings of this study are available in the supplementary material of this article.
