# Supplementary material for: An Apple Susceptibility‐Related LRR Receptor‐Like Kinase MdRLKT21 Activates Plant Immunity by Hijacking a Trans‐Kingdom Fungal microRNA‐Like RNA
Source: Adv Sci (Weinh). 2025 Aug 4;12(38):e17426. doi: 10.1002/advs.202417426 (PMC12520543; doi:10.1002/advs.202417426)
Supplement: Supplementary file 1 — Supporting Information [file ADVS-12-e17426-s002.docx]

**An apple susceptibility-related LRR receptor-like kinase MdRLKT21 activates plant immunity by hijacking a trans-kingdom fungal microRNA-like RNA**

**Authors:**

*Mingliang Lei^1^, Jian Zhang^1^, Jie Wang^1^, Chengyu Gao^1^, Yanting He^1^, Runze Tian^1^, Hao Feng^1^*, Lili Huang^1,2^**

**Affiliation:**

^1^State Key Laboratory for Crop Stress Resistance and High-Efficiency Production, College of Plant Protection, Northwest A&F University, Yangling, Shaanxi 712100, China.

^2^State Key Laboratory of Desert and Oasis Ecology, Key Laboratory of Ecological Safety and Sustainable Development in Arid Lands, Xinjiang Institute of Ecology and Geography, Chinese Academy of Sciences, Urumqi 830011, China.

***Corresponding authors:**

Hao Feng

E-mail: xiaosong04005@163.com

Lili Huang

E-mail: huanglili@nwsuaf.edu.cn

**Figure S1.** Bioinformatic analysis of MdRLKT21. a) Conserved domain analysis of MdRLKT21. b) Multiple protein sequence alignment of MdRLKT21. c) Phylogenetic analysis of MdRLKT21.

**Figure S2.** Subcellular localization of MdRLKT21. a) Transiently expressed MdRLKT21-GFP and green fluorescent protein (GFP) control in *N. benthamiana*. Fluorescence was imaged at 48 hpi using laser scanning confocal microscopy. Membrane protein TaWPI6 (mCherry-tagged) was used as the plasma membrane location marker. The white arrow indicates the primary fluorescence collection regions. Bar, 20 μm. b) Fluorescence intensity profiles of MdRLKT21-GFP, GFP control, and TaWPI6-mCherry were analysed using ImageJ software. c) Western blotting analysis of MdRLKT21-GFP and GFP control proteins using antibody anti-GFP.

**Figure S3.** The transient expression of *MdRLKT21* inhibits plant immunity in *N. benthamiana*. a,b) The transient expression of *MdRLKT21* significantly reduced resistance to *S. sclerotiorum* in *N. benthamiana*. Photographs were taken at 24 h post *S. sclerotiorum* inoculation. Lesion areas were measured using ImageJ software. Mean ± SD values were calculated from at least three independent biological replicates. c) Western blotting analysis of MdRLKT21-GFP and GFP control proteins using antibody anti-GFP. The asterisk indicates the position of MdRLKT21 protein. Ponceau S was used as the loading control. d) The transient expression of *MdRLKT21* decreased the expression levels of defence-related genes. *NbEF1α* was used as the inner reference gene. Mean ± SD were calculated from three biological replicates. e,f) The transient expression of *MdRLKT21* decreased reactive oxygen species (ROS) accumulation at 24 h post *S. sclerotiorum* inoculation. Bar, 50 μm. ImageJ software was used to quantify ROS accumulation per microscopic picture. Relative ROS accumulation values were normalized with the mean of empty vector (EV) control. Mean ± SD values were calculated from at least three independent biological replicates. g,h) The transient expression of *MdRLKT21* decreased callose deposition at 24 h post *S. sclerotiorum* inoculation. Bar, 100 μm. ImageJ software was used to quantify callose deposition per microscopic picture. Relative callose deposition values were normalized with the mean of empty vector (EV) control. Mean ± SD values were calculated from at three independent biological replicates. Statistical analyses were performed using two-tailed t-test (***P* < 0.01).

**Figure S4.** Oxidative burst in *N. benthamiana* expressing *MdRLKT21* and GFP was measured after treatment with 1μM flg22, 5μM COS, and water. ROS production was represented by total RLU. Mean ± SD values were calculated from three independent biological replicates. Different lowercase letters indicate the statistically significant differences (*P* < 0.05, one-way analysis of variance (ANOVA) and Tukey’s multiple comparison test).

**Figure** **S5.** Stable overexpression of *MdRLKT21* reduces apple calli resistance against *V. mali*. a) Identification of *MdRLKT21*-OE transgenic apple calli by DNA detection. b) Relative expression of *MdRLKT21* in stable transgenic apple calli. *MdEF1α* was used as the inner reference gene. Mean ± standard deviation (SD) values were calculated from three biological replicates. c,d) Stable overexpression of *MdRLKT21* reduced resistance to *V. mali* in apple calli. Photographs were taken at 40 h post *V. mali* inoculation. Lesion areas were measured using ImageJ software. Bar, 5 mm. Mean ± SD values were calculated from three independent biological replicates. Different lowercase letters indicate the statistically significant differences (*P* < 0.05, one-way analysis of variance (ANOVA) and Tukey’s multiple comparison test).

**Figure S6.** Identification of *MdRLKT21*-OE transgenic apple tissue culture seedlings by DNA detection. M, DL5000 DNA Marker; N, negative control; WT, nontransformed wild type; V, *MdRLKT21*-OE vector; OE-4, *MdRLKT21*-OE-4 transgenic apple line.

**Figure S7.** Stable overexpression of *MdRLKT21* decreases the expression levels of apple defence-related genes. *MdEF1α* was used as the inner reference gene. Mean ± SD were calculated from three biological replicates. Statistical analyses were performed using two-tailed t-test (***P* < 0.01).

**Figure S8.** Light and transmission electron micrographs of cross-sections of WT and *MdRLKT21*-OE transgenic apple leaves at 36 h post *V. mali* inoculation. EP, epidermis; HC, host cell; H, hyphae; HCW, host cell wall; Chl, chloroplast.

**Figure S9.** Transient silencing of *MdRLKT21* enhances resistance to *V. mali* in apple leaves. a) Relative expression levels of *MdRLKT21*. *MdEF1α* was used as the inner reference gene. Mean ± standard deviation (SD) values were calculated from three biological replicates. b,c) The transient silencing of *MdRLKT21* significantly enhanced apple resistance against *V. mali*. Photographs were taken at 36 h post *V. mali* inoculation. Lesion areas were measured using ImageJ software. Bar, 5 mm. Mean ± SD values were calculated from at least 18 replicates of three independent biological experiments. d) Transient silencing of *MdRLKT21* increased the expression levels of apple defence-related genes. *MdEF1α* was used as the inner reference gene. Mean ± SD were calculated from three biological replicates. Differences were assessed by two-tailed t-test (**P* < 0.05, ***P* < 0.01).

**Figure S10.** Identification of stable silencing apple calli of *MdRLKT21*. a) Identification of *MdRLKT21*-RNAi transgenic apple calli by DNA detection. b) Identification of *MdRLKT21*-RNAi transgenic apple calli by western blotting.

**Figure S11.** Identification of stable silencing apple seedlings of *MdRLKT21* and the expression pattern of *MdRLKT21* during *V. mali* infection. a) Identification of *MdRLKT21*-RNAi transgenic apple seedlings by DNA detection. b) Identification of *MdRLKT21*-RNAi transgenic apple seedlings by western blotting. c) Identification of *MdRLKT21*-RNAi transgenic apple seedlings by fluorescent labeling. Bar, 100 μm. d) The expression of *MdRLKT21* in the *V. mali*-susceptible late-ripening apple cultivar 'Qincui' after inoculation with *V. mali*. *MdEF1α* was used as the inner reference gene. Mean ± SD values were calculated from three independent biological replicates. Differences were assessed by two-tailed t-test (***P* < 0.01).

**Figure S12.** The expression of *MdRLKT21* is regulated by *Vm*-milR1 at the posttranscriptional level. a) *MdRLKT21* displayed reduced transcript levels during *V. mali* wild-type (WT) infection. b) The transcript levels of *MdRLKT21* exhibited no discernible difference during *Vm*-milR1 deletion mutant (ΔR1-55) infection. c) The expression levels of *MdRLKT21* in apple leaves during infection of WT and ΔR1-55 at 6 hpi. Error bars indicate the standard deviation (SD) values from three independent biological replicates. Statistical analyses were performed with the two-tailed t-test (**P* < 0.05, ***P* < 0.01, NS, not significant).

**Figure S13.** *Vm*-milR1 suppresses the expression of *MdRLKT21* in *N. benthamiana* by coinjection. a) *MdRLKT21* target site fused GFP was transiently expressed in *N. benthamiana* leaves with *Vm*-milR1 or control milRNA (*Vm*-milR16). Green fluorescent protein (GFP) fluorescence was observed at 48 h after agroinfiltration with a laser scanning confocal microscopy using the same parameter settings. Scale bar, 20 μm. b) GFP intensity was determined in at least three independent experiments. Relative GFP intensity was normalized to the mean of GFP intensity of MdRLKT21-fused GFP. Error bars indicate the SD values. Different lowercase letters indicate the statistically significant differences (*P* < 0.05, one-way analysis of variance (ANOVA) and Tukey’s multiple comparison test). c) Fused GFP was detected by western blotting. Ponceau S was used as the loading control. Similar results were obtained from three biological replicates.

**Figure S14.** a) The diagram of generation of *Vm*-milR1 target sites fused GFP construction. b) Alignment of *MdRLKT21* and *MdRLKT21* mutated version (*MdRLKT1*-m) with *Vm*-milR1.

**Figure S15.** The prediction of binding efficiency between *Vm*-milR1 and the apple receptor-like kinase genes *MdRLKT1*, *MdRLKT2*, and *MdRLKT21* based on _PS_RNAT_ARGET_.

**Figure S16.** RT-PCR was used to detect the expression level of *Vm*-milR1. 5S rRNA was used as the internal control. Similar results were obtained from three biological replicates.

**Figure** **S17.** a) Luciferase activity analysis of MdRLKT1-LUC in *N. benthamiana* leaves were infiltrated the vectors MdRLKT1-LUC, MdRLKT1-LUC/MdRLKT21-GFP, MdRLKT1-LUC/*Vm*-milR1, and MdRLKT1-LUC/MdRLKT21-GFP/*Vm*-milR1. b) Luciferase activity analysis of MdRLKT2-LUC in *N. benthamiana* leaves were infiltrated the vectors MdRLKT2-LUC, MdRLKT2-LUC/MdRLKT21-GFP, MdRLKT2-LUC/*Vm*-milR1, and MdRLKT2-LUC/MdRLKT21-GFP/*Vm*-milR1.

The target sites of MdRLKT1, MdRLKT2, and MdRLKT21 were fused with LUC and GFP, respectively. Different lowercase letters indicate the statistically significant differences (*P* < 0.05, one-way analysis of variance (ANOVA) and Tukey’s multiple comparison test). Similar results were obtained from three biological replicates.

**Figure S18.** Relative expression levels of *MdRLKT1* and *MdRLKT2* in *MdRLKT21*-overexpressed transgenic apple at 36 h post *V. mali* inoculation. *MdEF1α* was used as the inner reference gene. Mean ± SD were calculated from three biological replicates. Statistical analyses were performed using two-tailed t-test (**P* < 0.05, ***P* < 0.01).

**Figure S19.** The prediction of binding efficiency between *Vm*-milR1 and the pear receptor-like kinase genes *PbRLKT2* and *PbRLKT21* based on _PS_RNAT_ARGET_.

**Figure S20.** Confirmation of the interaction between MdRLKT21 and MdRFP1 by a luciferase complementation imaging (LCI) assays, the combinations of GFP-nLUC with cLUC-GFP, MdRLKT21-nLUC with cLUC-GFP, and GFP-nLUC with cLUC-MdRFP1 served as the negative control.

**Figure S21.** Structural basis of MdRLKT21 binding to MdRFP1. The three-dimensional structural models of MdRLKT21 (yellow) and MdRFP1 (blue) were generated using the SWISS-MODEL server. The MdRLKT21 receptor and MdRFP1 ligand were respectively docked using the HDOCK server. The optimal docking model was selected and analyzed for dimerization interface residues and interaction. Key interacting residues of MdRLKT21 and MdRFP1 are highlighted in red. The dashed lines represent the interaction forces between MdRLKT21 and MdRFP1.

**Figure S22.** The GST-MdRLKT21-KD and GST-MdRFP1-His recombinant proteins were obtained by prokaryotic expression. The red asterisks indicate the position of GST-MdRLKT21-KD and GST-MdRFP1-His proteins.

**Figure S23.** The expression pattern of *MdRFP1* during *V. mali* infection. *MdEF1α* was used as the inner reference gene. Mean ± SD values were calculated from three independent biological replicates. Differences were assessed by two-tailed t-test (**P* < 0.05, ***P* < 0.01).

**Figure S24.** Subcellular localization of MdRFP1. a) Transiently expressed MdRFP1-GFP and GFP control in *N. benthamiana*. Fluorescence was imaged at 48 hpi using laser scanning confocal microscopy. Membrane protein TaWPI6 was used to mark the plasma membrane. Bar, 20 μm. b) Western blotting analysis of MdRFP1-GFP and GFP control proteins using antibody anti-GFP.

**Figure S25.** The GST-MdRFP1and GST recombinant proteins were obtained by prokaryotic expression. The red asterisks indicate the position of GST-MdRFP1 and GST proteins.

**Figure S26.** Identification of stable overexpression apple calli of *MdRFP1*. a) Identification of *MdRFP1*-OE transgenic apple calli by DNA detection. b) Identification of *MdRFP1*-OE transgenic apple calli by western blotting.

**Figure S27.** Overexpression of *MdRFP1* in stable transgenic apple calli increased O^2−^ contents and O^2−^ production rate. a) O^2−^ contents of WT, *MdRFP1*-OE-3, and *MdRFP1*-OE-8 apple calli at 3 dpi. b) O^2−^ production rate of WT, *MdRFP1*-OE-3, and *MdRFP1*-OE-8 apple calli at 3 dpi. Mean ± SD values were calculated from three independent biological replicates. Different lowercase letters indicate the statistically significant differences (*P* < 0.05, one-way analysis of variance (ANOVA) and Tukey’s multiple comparison test).

**Figure S28.** Transient silencing of *MdRFP1* reduces resistance to *V. mali* in apple leaves. a) Relative expression levels of *MdRFP1*. *MdEF1α* was used as the inner reference gene. Mean ± standard deviation (SD) values were calculated from three biological replicates. b,c) The transient silencing of *MdRFP1* significantly reduced apple resistance against *V. mali*. Photographs were taken at 36 h post *V. mali* inoculation. Lesion areas were measured using ImageJ software. Bar, 5 mm. Mean ± SD values were calculated from at least 35 replicates of three independent biological experiments. d,e) Apple leaves silenced with *MdRFP1* showed decreased ROS accumulation at 36 h post *V. mali* inoculation. Bar, 200 μm. Relative ROS accumulation values were normalized with the mean of empty vector (EV) control. Mean ± SD values were calculated from at least three independent biological replicates. Differences were assessed by two-tailed t-test (***P* < 0.01).

**Figure S29.** Relative expression levels of *MdRFP1* and *MdRLKT21* in apple leaves expressing the vectors EV, *MdRFP1*-OE, and *MdRFP1*/*MdRLKT21*-OE. a) The expression of *MdRFP1* in *MdRFP1*-OE and *MdRFP1*/*MdRLKT21*-OE leaves. b) The expression of *MdRLKT21* in *MdRFP1*/*MdRLKT21*-OE leaves. Mean ± standard deviation (SD) values were calculated from three biological replicates. Different lowercase letters indicate the statistically significant differences (*P* < 0.05, one-way analysis of variance (ANOVA) and Tukey’s multiple comparison test). Differences were assessed by two-tailed t-test (***P* < 0.01).

**Figure S30.** Relative expression of defense-related marker genes in apple leaves infiltrated with the vectors EV, *MdRFP1*-OE, and *MdRFP1*/*MdRLKT21*-OE. *MdEF1α* was used as the inner reference gene. Mean ± standard deviation (SD) values were calculated from three biological replicates. Different lowercase letters indicate the statistically significant differences (*P* < 0.05, one-way analysis of variance (ANOVA) and Tukey’s multiple comparison test).

**Figure S31.** The protein abundance of MdRFP1-mCherry in WT and *MdRLKT21*-RNAi-1/6/8 calli lines. The immunoblotting was performed with anti-mCherry antibody. MdActin was used as the loading control. The amounts of proteins were quantified using ImageJ software, and the protein levels of WT were set to 1.

**Figure S32.** MdRFP1 affects the protein stability of MdRLKT21-KD in *N. benthamiana*. Total proteins separately were extracted from *N. benthamiana* leaves expressing the MdRFP1-mCherry/MdRLKT21-KD-GFP and mCherry/MdRLKT21-KD-GFP with or without 50 μm MG132. The immunoblotting was performed with anti-GFP and anti-mCherry antibodies. Ponceau S was used as the loading control.

**Figure S33.** MdRFP1 ubiquitinates the MdRLKT21 protein *in vivo* and *in vitro*. a) The MdRLKT21-KD-His recombinant protein was obtained by prokaryotic expression. The red asterisk indicates the position of MdRLKT21-KD-His protein. b) MdRFP1 promoted the ubiquitination of MdRLKT21-KD-His protein *in vivo*. MdRLKT21-KD-His was immunoprecipitated with the anti-His antibody. The immunoprecipitated proteins were detected with anti-His and anti-Ubi antibodies. Input was detected using anti-Actin antibody. IP, immunoprecipitate; IB, immunoblot; Ubi, ubiquitin. c) Ubiquitination assay of MdRLKT21-KD-His by MdRFP1 *in vitro*. MdRLKT21-KD-His protein was incubated with GST-MdRFP1-His protein, E1, E2, ATP and ubiquitin *in vitro*. The ubiquitination of MdRLKT21-KD-His was detected by anti-His antibody. The GST-MdRFP1-His protein was also detected using anti-His antibody.

**Figure S34.** A schematic diagram for the MdRLKT21- MdRFP1 module in regulating immunity. MdRLKT21 interacts with and phosphorylates the resistance protein MdRFP1, resulting in decreased MdRFP1 stability through the ubiquitin pathway, thereby suppressing immune responses.
